# Highly Efficient Photothermal‐Catalytic Depolymerization of Polyester Fiber Enabled by a Phosphotungstate‐Based Palladium Single‐Atom Catalyst

**DOI:** 10.1002/smll.202505673

**Published:** 2025-09-15

**Authors:** Xin Li, Yiming Bu, Lu Jiang, Qining Fan, Yue You, Qi Han, Xiaokai Hu, Hongjun Yang, Joselito Macabuhay Razal, Christoper Hurren, Jingliang Li

**Affiliations:** ^1^ Institute for Frontier Materials Deakin University Waurn Ponds Campus Geelong Victoria 3216 Australia; ^2^ Future Industries Institute UniSA STEM University of South Australia Mawson Lakes Campus Adelaide SA 5095 Australia; ^3^ School of Science STEM College RMIT University Melbourne VIC 3000 Australia; ^4^ School of Mechanical and Electrical Engineering Guilin University of Electronic Technology Guilin 541004 China; ^5^ Key Laboratory for Green Processing & Application of New Textile Materials of the Education Ministry Wuhan Textile University Wuhan 430065 China

**Keywords:** local heating effect, photothermal depolymerization, polyester fiber, single‐atom catalyst

## Abstract

The chemical inertness and thermal stability of plastics contribute to their durability and stability across a wide range of applications. However, these same properties present significant challenges for their recycling under mild conditions. In this study, a novel photothermal catalytic system is developed featuring Pd single‐atom catalysts (SACs) anchored on activated carbon for the efficient and rapid photothermal depolymerization of polyethylene terephthalate (PET) by ethylene glycol (EG). Different from top‐down lighting used in other studies, side lighting is used to avoid the negative influence of EG evaporation on the photothermal conversion. By optimizing the structure of the catalyst, near complete PET decomposition within 4 h under a light intensity of 0.5 W cm^−2^ is achieved. Density functional theory (DFT) calculations reveal for the first time that the unique electronic structure of palladium (Pd) facilitates the nucleophilic attack of EG on PET. The strong coordination between the carbonyl oxygen in PET and the Pd significantly lowers the reaction energy barrier from 37.44 to 26.77 kcal mol^−1^. This study presents a promising strategy for designing efficient catalysts, offering a cost‐effective and sustainable solution for PET recycling.

## Graphical abstract

In this work, a novel phosphotungstate‐based single‐atom catalyst (SAC) supported on cost‐effective activated carbon is developed for the photothermal depolymerization of polyester fibers. The activated carbon efficiently harnesses solar energy to rapidly heat the reaction system, reaching 185 °C within 20 min, leading to complete depolymerization of polyester fibers in 4 h.

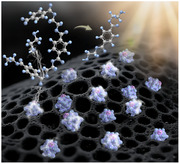

## 1 Introduction

The widespread use of plastics has led to persistent environmental pollution, posing a serious threat to global ecosystems. Discarded plastics are commonly found in oceans, lakes, rivers, soils, sediments and even the atmosphere, where they disrupt natural processes and harm biodiversity.^[^ ^1^, ^2^, ^3^, ^4^, ^5^ ^]^ Among these, polyethylene terephthalate (PET), a common type of polyester, is one of the most prevalent,^[^ ^6^, ^7^, ^8^, ^9^ ^]^ accounting for nearly 12% of total solid waste.^[^ ^10^, ^11^ ^]^ PET is the primary material of plastic bottle and serves as a primary raw material for the production of synthetic textile fibers. During washing, PET fibers release substantial amounts of microplastic particles, which pose a threat to aquatic life and ultimately enter the food chain, thereby impacting human health.^[^ ^12^ ^]^ Consequently, developing effective strategies for PET recycling is critical to mitigating plastic pollution.

Pyrolysis, hydrolysis and alcoholysis have been employed for PET depolymerization and recycling.^[^ ^6^, ^8^, ^13^, ^14^, ^15^ ^]^ Among them, alcoholysis of PET using ethylene glycol (EG) has attracted more research attention as it operates under mild conditions and atmospheric pressure. Furthermore, all the reactants and product are non‐toxic and non‐corrosive, making the process environmentally friendly.^[^ ^16^, ^17^, ^18^, ^19^, ^20^, ^21^ ^]^ Alcoholysis yields high‐purity monomer bis(2‐hydroxyethyl) terephthalate (BHET) and valuable oligomers (such as dimers, trimers and tetramers), which can be repurposed for manufacturing new PET,^[^ ^22^ ^]^ as well as other materials like epoxy resin (ER), vinyl ester resin (VER), and enhanced polyurethanes foams.^[^ ^23^, ^24^ ^]^ However, despite its advantages, alcoholysis still requires elevated temperatures ranging from 170 to 300 °C,^[^ ^6^ ^]^ leading to substantial energy consumption.

Photothermal conversion has emerged as a promising strategy to reduce the energy consumption of various reactions and processes.^[^ ^25^, ^26^ ^]^ Unlike conventional electrically powered thermal processes, photothermal process harnesses clean and abundant solar energy through a light‐absorbing material, offering a sustainable and efficient alternative. This technology has been successfully applied in water evaporation,^[^ ^27^, ^28^, ^29^ ^]^ CO_2_ reduction,^[^ ^30^, ^31^ ^]^ hydrogenation of CO_2_^[^ ^32^, ^33^ ^]^ and the degradation of waste plastics.^[^ ^34^, ^35^ ^]^ For example, silver nanoparticles embedded in low‐density polyethylene (LDPE) were used to create a photothermal effect that in combination with cobalt (II) stearate as a catalyst significantly accelerated LDPE degradation.^[^ ^36^ ^]^ Similarly, metal nanoparticles have been employed to induce photothermal degradation of poly (ethyl cyanoacrylate), producing graphene‐like and luminescent carbonaceous by‐products.^[^ ^37^ ^]^ A photothermal catalytic approach has also been developed to recover value‐added monomers from waste PET. For instance, polydopamine‐modified multi‐walled carbon nanotubes (CNTs‐) was used as solar absorbers, with choline phosphate as the catalyst, to enable PET depolymerization at a relatively low temperature of 150 °C.^[^ ^38^ ^]^ Based on this, a subsequent study reported complete PET conversion within 3 h of sunlight irradiation using a single‐site catalyst (SSC) based on Co–O_5_ coordination.^[^ ^39^ ^]^ Although this study has achieved significant progress in PET degradation, several key challenges remained. CNTs need to be functionalized to improve their dispersion in EG, which adds more cost to the already expensive CNTs. The potential toxicity of CNTs^[^ ^40^ ^]^ has also been a concern for their practical applications. In addition, SSCs offer limited tunability of their electronic properties of the active sites. Additionally, like other studies, the way of light irradiation needs to be optimized. The high temperature vaporizes the solvent which scatters and absorb light when light is irradiated top down to the reaction medium. This induces energy loss and hence necessitates the use of high light intensities.

Despite the promising progress in PET depolymerization via photothermal catalysis, the development of efficient catalysts remains in its early stages. There is a clear need for novel and cost‐effective catalysts to advance this field. Single atom catalysts (SACs), which feature isolated metal atoms dispersed on the surface of a support material,^[^ ^41^ ^]^ have gained significant attention due to their high catalytic efficiency and exceptional atomic utilization. Various methods, including atomic layer deposition,^[^ ^42^ ^]^ chemical reduction,^[^ ^43^ ^]^ and coprecipitation,^[^ ^44^ ^]^ have been used to synthesize SACs. Among these, coprecipitation stands out for its simplicity, low cost, and suitability for a broad range of metals. Among the many support materials used for SACs, homogeneous polyoxometalates (POMs) are particularly promising due to their well‐defined molecular structures and tunable properties.^[^ ^41^ ^]^ In contrast to traditional heterogeneous supports like metal oxides and carbon materials, which often rely on poorly controlled binding sites such as defects or doped regions, POMs offer uniform, well‐characterized coordination sites that can effectively anchor individual metal atoms. Although POM‐based SACs have demonstrated impressive performance for various chemical reactions, including CO oxidation,^[^ ^45^ ^]^ methane oxidation,^[^ ^46^ ^]^ and photocatalytic hydrogen production,^[^ ^47^ ^]^ their applications for plastic degradation remain unexplored.

In this study, phosphotungstic acid (PTA) is employed as a model POM to construct a SAC. Palladium is anchored onto PTA in the form of isolated atoms (Pd‐CsPTA), and the resulting complex is subsequently supported on activated carbon (AC), a photothermal material, to form Pd‐CsPTA/AC) for solar‐assisted catalytic depolymerization of PET. Although various homogeneous and heterogeneous catalysts, such as those based on Zn,^[^ ^48^, ^49^ ^]^ Fe^[^ ^50^, ^51^ ^]^ and Co,^[^ ^52^, ^53^ ^]^ have been developed to reduce the reaction temperature, their catalytic activities remain insufficient. Fe and Co catalysts are limited by low selectivity and complex product profiles,^[^ ^52^ ^]^ while Zn catalysts suffer from insufficient activity and typically require organic co‐catalysts to be effective.^[^ ^54^ ^]^ Although palladium is relatively expensive, its excellent electronic structure facilitates better dispersion on surface of the support, thereby significantly enhancing catalytic activity and atomic utilization.^[^ ^44^ ^]^ Moreover, its favorable redox properties (i.e., reversible transformation between Pd⁰ and Pd^2^⁺) are expected to enable stable electronic regulation during the reaction, which can promote the cleavage of C─O and C═O to accelerate the depolymerization of PET.

To enhance photothermal efficiency, side lighting was used to minimize the adverse effect of EG vapor on light exposure to the reaction medium. This adjustment enabled the system to more rapidly reach the required temperature for efficient depolymerization. Density functional theory (DFT) calculations were conducted to investigate the catalyst's active site and elucidate the underlying reaction mechanism. This work introduces a novel and efficient SAC system for green, low‐cost, solar‐driven PET depolymerization, offering a promising pathway for sustainable PET recycling.

## 2 Results and Discussion

### 2.1 Synthesis and Characterization of Pd‐CsPTA and Pd‐CsPTA/AC

**Figure 1:**
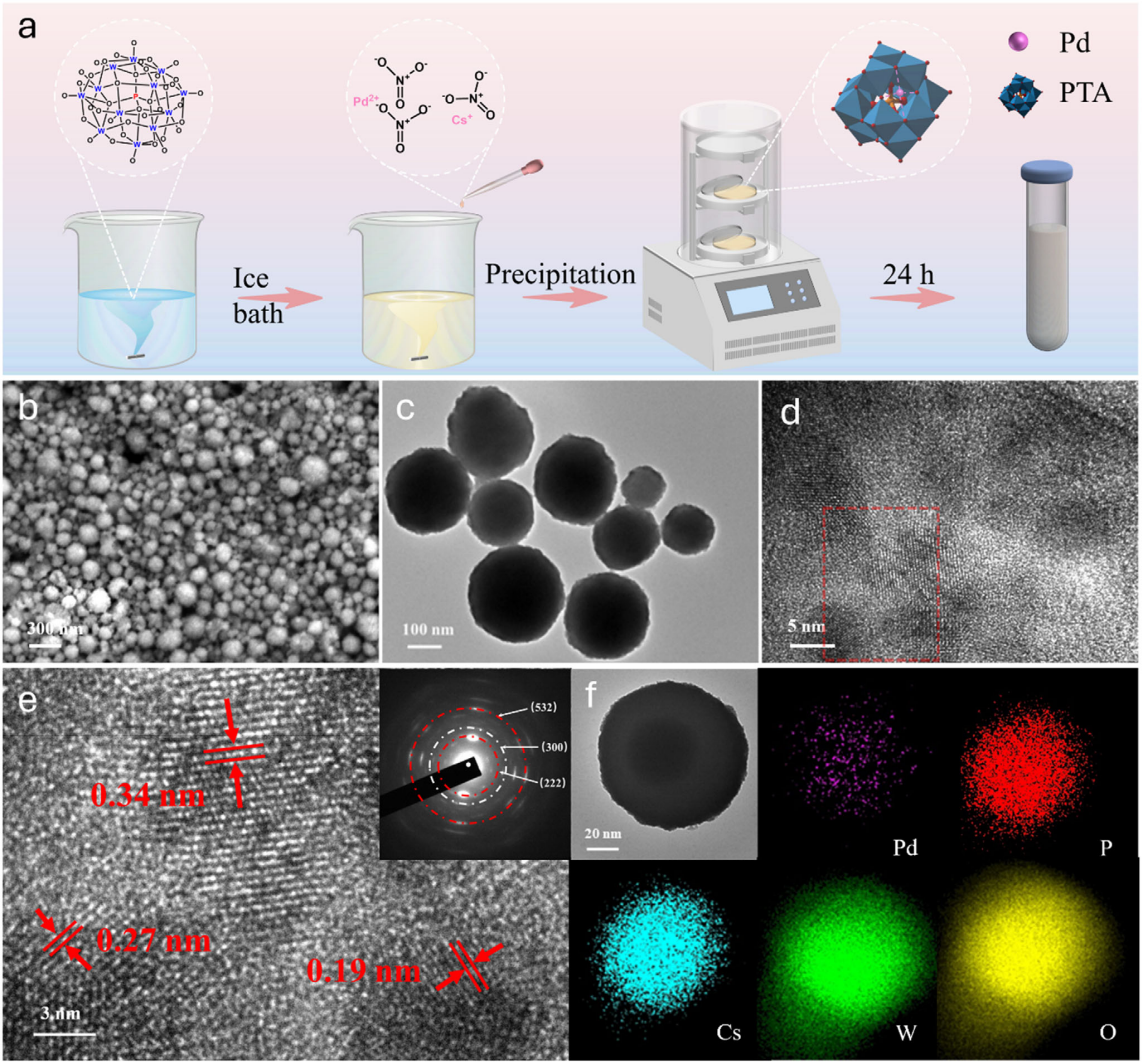
Preparation and morphological characterization of phosphotungstate‐based palladium SACs. a) Schematic illustration for synthesis of Pd‐CsPTA catalyst. b,c) SEM and TEM images of Pd‐CsPTA particles. d,e) High‐resolution TEM images, and f) EDS element mapping of Pd‐CsPTA particles.

Pd‐CsPTA was synthesized by co‐precipitation of palladium nitrate, cesium nitrate, and PTA solution in an ice bath (**Figure** **1**a). The size, crystal structure, and elemental distribution of the Pd‐CsPTA were characterized by scanning electron microscopy (SEM), transmission electron microscopy (TEM) and energy‐dispersive X‐ray spectroscopy (EDS). The SEM and TEM images (Figure 1b,c) show that spherical Pd‐CsPTA particles with diameters ranging from 100–350 nm formed. The three unique lattice spacings in the high‐resolution TEM image (Figure 1d,e) indicate that the Pd‐CsPTA particles are polycrystalline. EDS mapping shows a quite uniform distribution of Pd on the particles (Figure 1f).

**Figure 2:**
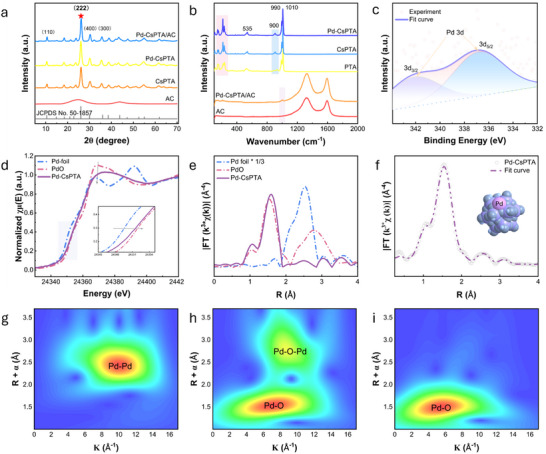
Structural characterization of phosphotungstate‐based palladium SACs. a) XRD, b) Raman and c) XPS spectra of the catalyst with reference materials. d) Normalized XANES and e) K^3^‐weight FT‐EXAFS spectra of catalysts with Pd‐foil and PdO. f) FT‐EXAFS fitting curve of Pd‐CsPTA. g–i) WT‐EXAFS plots of Pd‐foil, PdO and Pd‐CsPTA.

In the XRD pattern of Pd‐CsPTA (**Figure** **2**a), the sharp characteristic peak at 26.5° is attributed to the special Keggin structure of PTA, which reflects its highly ordered crystalline framework.^[^ ^44^ ^]^ This peak corresponds to the 0.34 nm lattice spacing observed in the TEM image (Figure 1e). The Raman spectra of PTA and CsPTA showed that the replacement of protons by cesium affected the electron distribution within the W═O and W──O──W bonds, causing a shift of the peak at 900 cm^−1^ (Figure 2b). The typical low‐frequency vibration region of metal–oxygen bond was observed at 150–300 cm^−1^, with W──O──W bending vibrations occurring in the 210–240 cm^−1^ region. Upon Pd coordination with oxygen, the metal–oxygen bond vibrations were intensified, accompanied by the diminishing of the W──O──W vibrations and shift of the characteristic peak to lower frequencies. X‐ray photoelectron spectroscopy (XPS) was used to investigate the valence states of Pd species on the Pd‐CsPTA. The spectrum showed two symmetric peaks at 341.8 and 336.2 eV, corresponding to the Pd 3d_3/2_ and Pd 3d_5/2_ (Figure 2c), respectively. The position of the Pd 3d_5/2_ peak was commonly used to determine the oxidation state of Pd.^[^ ^44^ ^]^ According to a previous work,^[^ ^55^ ^]^ Pd metal was characterized by a peak at 335 eV, whereas oxidized Pd exhibited the peak at 336.7 eV. The experimentally fitted peak was located between these two values, indicating that Pd was oxidized, though with a valence sate lower than +2. The absence of a characteristic XRD peak of Pd (111) and the XPS results indicated that Pd was dispersed on CsPTA in single atoms.

To better understand the local coordination and electronic structure of Pd species and to verify its atomic dispersion on CsPTA particles, X‐ray absorption near‐edge structure (XANES) and extended X‐ray absorption fine structure (EXAFS) analysis were conducted at the Pd K‐edge. As shown in the XANES spectra (Figure 2d), the absorption edge of Pd in Pd‐CsPTA appeared at 24357.3 eV, which is between those of Pd foil and PdO, further evidence that the valence of Pd was between 0 and +2. By referencing the absorption edge positions of Pd (0) and Pd (II), fitting results (Figure S1, Supporting Information) indicated the Pd valence state of +1.725 in Pd‐CsPTA. Additionally, the white line intensity of Pd‐CsPTA at 24365 eV was higher than that of Pd foil, further indicating that Pd was in an oxidized state, consistent with the XPS results. In the k^3^‐weighted EXAFS Fourier transform of Pd‐CsPTA (Figure 2e), only a Pd──O scattering path at 1.5 Å was detected with absence of a Pd──Pd shell at 2.5 Å. This strongly confirmed that Pd was in the form of isolated atoms. The wavelet transforms of the EXAFS spectrum with an energy term showed a maximum intensity for Pd‐CsPTA at *K* = 6 Å^−1^ and *R* = 1.5 Å. It was attributed to the Pd──O path, which is similar to the Pd──O path (*K* = 7 Å^−1^, *R* = 1.5 Å), but different from the Pd──O──Pd path (*K* = 9.5 Å^−1^, *R* = 2.7 Å) in PdO (Figure 2g–i). The fitting results (Figure 2f, Figure S2 and Table S1, Supporting Information) revealed that each Pd atom was coordinated with four oxygen atoms at about 2 Å. This means every Pd atom occupied a fourfold hollow site on the Cs‐PTA. Furthermore, DFT calculations revealed that the lowest binding energy (‐64.22 kcal mol^−1^) was obtained when the Pd was loaded at the oxygen fourfold hollow site, indicating this site provided the most stable binding environment for Pd (Figure S3 and Table S2, Supporting Information). This is consistent with the EXAFS data, confirming the Pd‐O_4_ structure.

Although PTA exhibited excellent thermal stability and provided coordination sites for single‐atom Pd loading, its limited photothermal conversion capability means that it must be integrated with an excellent photothermal material.^[^ ^41^ ^]^ AC, a porous carbonaceous material (Figure S4, Supporting Information) that can be derived from cheap resources such as biomass, features low cost and excellent renewability. Its high surface activity, adsorption capabilities and light absorption also make it an ideal catalyst support and photothermal material. Compared to other prominent nanomaterials including carbon nanotube (CNT), graphene oxide (GO) and Mxene, AC exhibited similar photothermal conversion efficiency (Figure S5a, Supporting Information) while it adsorbed more PTA (Figure S5b, Supporting Information). Most importantly, AC can be stably dispersed in EG for more than 3 d (Figure S6, Supporting Information), while the other nanomaterials settled in EG.

**Figure 3:**
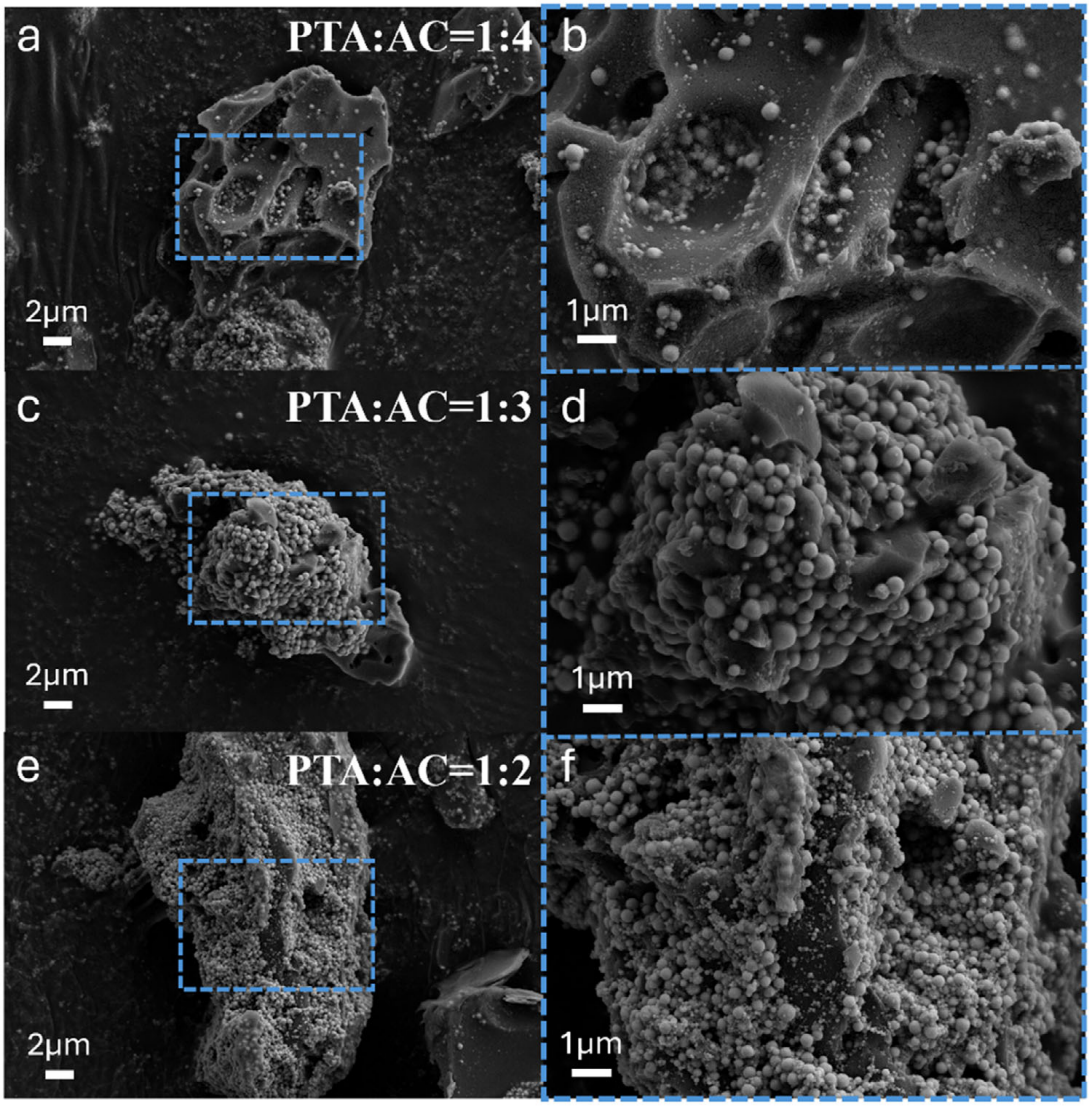
SEM images of Pd‐CsPTA/AC synthesized with different ratios of PTA and AC. a,b) PTA:AC = 1:4. c,d) PTA:AC = 1:3. e,f) PTA:AC = 1:2.

The procedure for loading Pd‐CsPTA on AC is similar to that shown in Figure 1a, except that AC particles were presented in the precursor PTA aqueous solution in the first step. The ratio of AC to PTA directly affected the loading degree of Pd‐CsPTA on AC, its photothermal conversion properties and catalytic activity. As shown in **Figure** **3**c,d, when PTA:AC was 1:3, Pd‐CsPTA exhibited uniform size and well‐formed structure, with even distribution on AC. Although increasing the amount of PTA reduced the size of Pd‐CsPTA, which could be due to enhanced nucleation of PTA on AC, it also promoted the aggregation of Pd‐CsPTA (Figure 3e,f). EDS mapping (Figure S7, Supporting Information) of Pd‐CsPTA prepared at the optimal PTA:AC ratio of 1:3 clearly showed even distribution of the elements P and Pd. In comparison to pure AC, Pd‐CsPTA/AC exhibited a decrease in both surface area and pore volume but demonstrated significantly enhanced thermal stability (Figure S8a–c and Table S3, Supporting Information). This might be due to alteration of the surface properties of AC by Pd‐CsPTA, thereby influencing its thermal decomposition at high temperatures. Fourier transform infrared (FTIR), XRD pattern and Raman spectroscopy further confirmed the presence of characteristic peaks of the Keggin structure of PTA on Pd‐CsPTA/AC, and the crystalline structure of the catalysts maintained intact during the loading process (Figure 2a,b and Figure S8d, Supporting Information). The broadening of the FTIR characteristic peaks was a consequence of light scattering caused by the pore structure.

### 2.2 Photothermal Conversion

**Figure 4:**
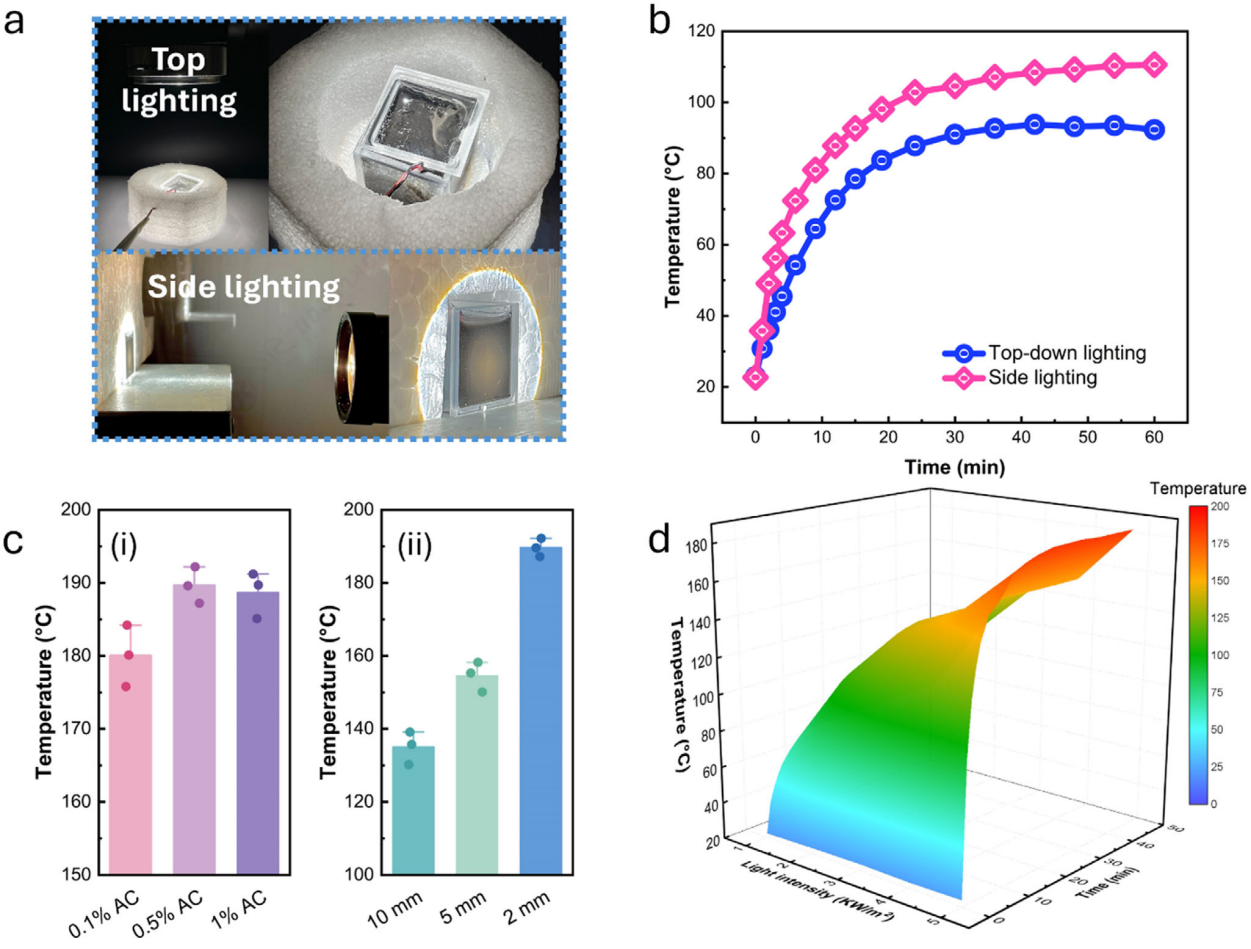
Photothermal conversion efficiency. a) The images of top‐down and side lighting photothermal systems. b) Temperature curves of top‐down lighting and side lighting with simulated sunlight (0.5 W cm^−2^). c) Temperature of EG after 15 min of illumination with (i) different AC ratios and (ii) optical path. d) Temperature of EG over time under different light intensities.

To achieve the required depolymerization temperature of PET in EG while minimizing solar energy consumption, optimization of angle of incidence of light and adjustment of Pd‐CsPTA/AC content were needed. As the temperature required for PET depolymerization is close to the boiling point of EG and EG may absorb water due to its hydrophilicity, the evaporation of water and EG and their condensation on the cover of the container may scatter or absorb light to reduce reach of light to the AC particles, if light is irradiated to the top of the vessel (i.e., top‐down lighting). This comprises the photothermal efficiency. To address this problem, this work introduced an innovative strategy by positioning the light beam perpendicular to the EG evaporation direction (i.e., side lighting) (**Figure** **4**a). At the same light intensity, side lighting efficiently mitigated the negative effect of EG/water vapor on the light absorption of photothermal materials, thereby accelerating the temperature rise and achieving a higher equilibrium temperature (Figure 4b). When the optical path of the reaction vessel was 2 mm (Figure S9, Supporting Information), the temperature of the EG dispersion reached approximately 185 °C within 20 min (Figure 4c‐ii). Furthermore, when the concentration of Pd‐CsPTA/AC was above 0.5%, the photothermal conversion efficiency was not significantly improved (Figure 4c‐i). High concentrations of AC could induce multiple light scattering effects, thereby weakening effective light absorption and reducing overall photothermal conversion efficiency. Therefore, side lighting, vessel with a 2 mm optical path and 0.5% Pd‐CsPTA/AC were chosen for further investigation. The temperature changes over time under different light intensities were given in Figure 4d.

For a comprehensive evaluation of the photothermal conversion efficiency, the temperature increase was compared with previously reported photothermal systems. The result revealed that the side lighting enabled more efficient photothermal conversion, evidenced by the achievement of a temperature (e.g., 190 °C) under a lower light intensity (Figure S10, Supporting Information). Although the maximum temperature slightly declined after several cycles, the heating and cooling rates were consistent (Figure S11, Supporting Information), indicating a stable photothermal conversion efficiency.

### 2.3 Photothermal Depolymerization of PET by Pd‐CsPTA/AC

The photothermal catalytic activity of Pd‐CsPTA/AC for the glycolysis of PET was evaluated. As an environmentally friendly and safe glycolysis agent, EG can effectively break the ester bonds of PET under relatively mild conditions, producing oligomers and monomers (Figures S12 and S13, Supporting Information). These products can be directly used to produce new PET or other polyester materials.

The photothermal depolymerization product was confirmed using high‐resolution liquid chromatography–tandem mass spectrometry (HR‐LC‐MS/MS) and nuclear magnetic resonance spectroscopy (NMR). HR‐LC‐MS/MS analysis revealed that the experimental product and the BHET standard exhibited identical main ion peaks at m/z = 255, along with consistent adduct (m/z = 277) and fragment (m/z = 237) peaks. The similarity in relative abundance further confirms the identity of the product as BHET. In the ^1^H NMR spectrum (Figure S15, Supporting Information), the signal at 8.10 ppm corresponds to aromatic protons, and the peak at 4.90 ppm is attributed to the hydroxyl proton. The peaks at 4.30 ppm and 3.70 ppm are the characteristics peaks of the methylene hydrogen of COOCH_2_─ and ─CH_2_OH, respectively. There are no signals other than BHET in the spectrum, indicating that the product is of high purity.

**Figure 5:**
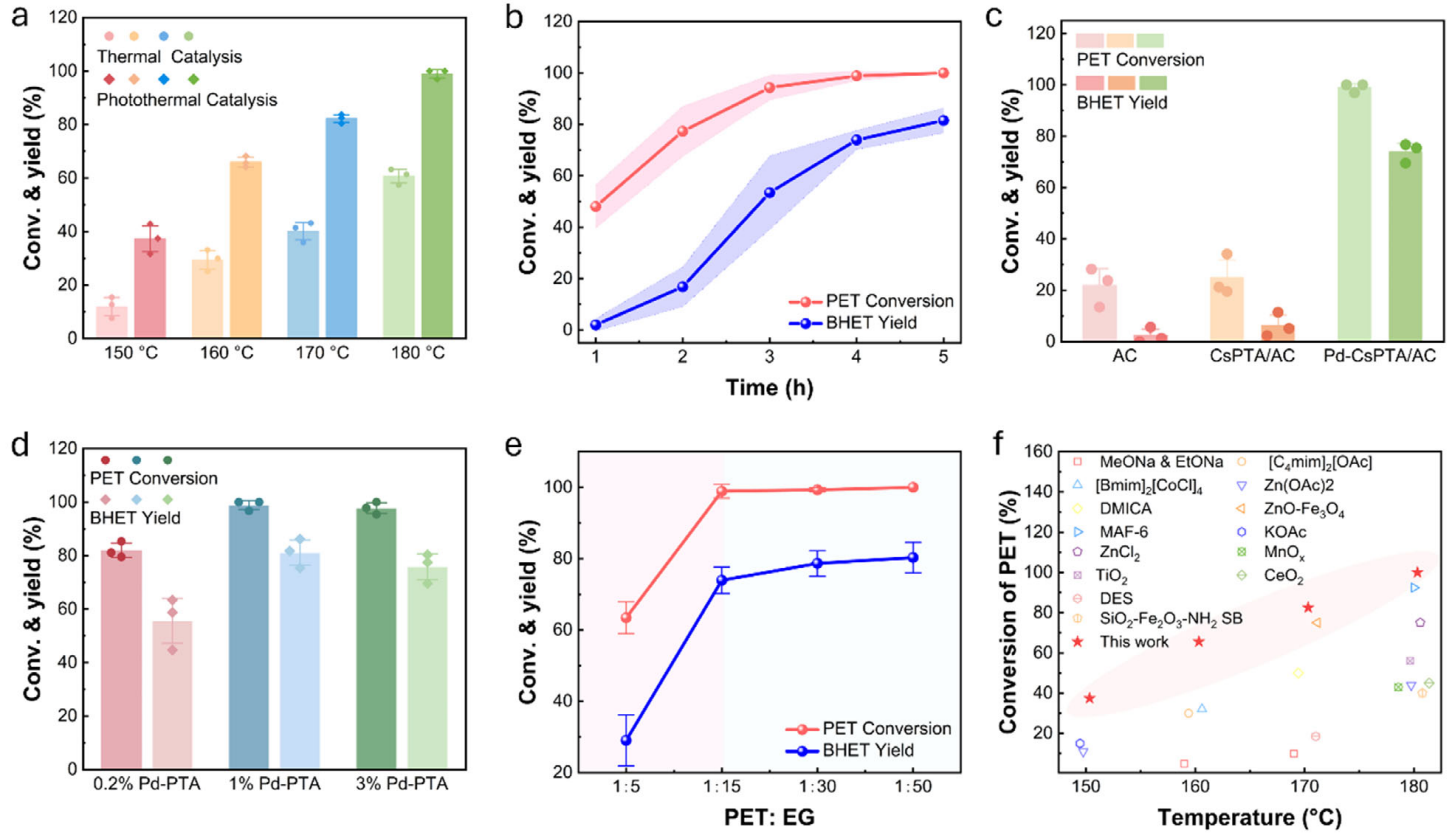
Optimization of PET depolymerization. a) comparison of efficiency of thermal and photothermal catalysis for depolymerization, b) Effects of photothermal reaction time (180 °C), c) effects of the catalyst, d) effect of catalyst loading, e) ratio of PET to EG on the conversion of PET and yield of BHET, and f) Comparison with reported catalysts for PET depolymerization.

Under thermal catalysis (i.e., electrical heating instead of photothermal heating), BHET was not obtained at 150 °C (**Figure** **5**a). In contrast, photothermal catalytic depolymerization at this temperature achieved PET conversion equivalent to thermal catalysis at 170 °C, highlighting the superior efficiency of photothermal catalysis. At 180 °C, PET was completely decomposed within 4 h, and the BHET yield reached around 80% after 5 h (Figure 5b). Furthermore, to evaluate the practical productivity of the reaction system, the spatio‐temporal yield (STY) was calculated and found to be approximately 60 g L^−1^ h^−1^. To identify the active site of Pd‐CsPTA/AC, PET depolymerization was assessed using AC, CsPTA/AC, or Pd‐CsPTA/AC. In the absence of Pd, the depolymerization efficiency of CsPTA/AC was comparable to that of AC alone (Figure 5c). In contrast, the presence of Pd led to a fourfold increase in the depolymerization rate and a substantial improvement in BHET yield (Figure 5c), indicating that Pd was the principal active center. These findings underscored the necessity of optimizing Pd loading. Further experiments indicated that a 1% Pd loading achieved nearly the same performance as a 3% loading (Figure 5d), likely due to Pd atom aggregation at a higher loading, which decreased dispersion and the availability of active sites.^[^ ^56^ ^]^ As PET glycolysis is an equilibrium reaction, an increase in the ratio of EG to PET favours the forward reaction. When the ratio of EG to PET was increased from 5:1 to 15:1 (corresponding to PET/EG ratio of 1:5 and 1:15, respectively), the depolymerization rate of PET and the yield of BHET were significantly improved from 63.43% to 98.90% and from 29.03% to 73.93%, respectively (Figure 5e). A further increase of EG did not lead to significant increase in the rate and yield. The catalytic activity also markedly exceeded most documented results, particularly at lower temperatures around 150–160 °C (Figure 5f and Table S4, Supporting Information).

To evaluate the reusability and performance stability of Pd‐CsPTA/AC, multiple cycling experiments were conducted. The recovery rate of Pd‐CsPTA/AC after catalytic depolymerization was approximately 85%. The PET depolymerization rate remained at 95% of its initial value after 5 cycles, but the BHET yield declined to 80% of the original value (73.6%) (Figure S16, Supporting Information). The catalyst's structure and morphology after reaction was conducted. As shown in Figure S17a (Supporting Information), the crystalline structure of the catalyst remained the same. XANES analysis (Figure S17b, Supporting Information) showed that, after catalysis, the Pd absorption edge shifted toward lower energies, indicative of a slight increase in local electron density. Notably, the white‐line intensity (the shaded area in Figure S17b, Supporting Information) remained essentially unchanged, implying that the electronic configuration and coordination environment of Pd were preserved during the reaction. Such electronic stability was corroborated by the EXAFS spectra (Figure S17c, Supporting Information), in which the after‐reaction spectrum remained dominated by the Pd─O peak at 1.6 Å, indicating that the Pd species remained atomically dispersed after reaction. However, partial leaching of Pd from the activated carbon (AC) support occurred (Figure S17d, Supporting Information), explaining the slight reduction in depolymerization efficiency. Despite this, a combination of structural stability and the degradation performance results confirmed the excellent recyclability of the catalyst.

### 2.4 Mechanism of Photothermal PET Depolymerization

#### 2.4.1 Localized Heating Effect Enhanced Depolymerization Efficiency

In contrast to thermal catalysis, which generally relies on electrical power to generate thermal energy, photothermal catalysis can derive its thermal energy from renewable solar energy. However, apart from thermal catalysis, photocatalytic degradation by UV light is a general method to decompose a material. To understand if the PET depolymerization was through thermal or photocatalytic pathway, the UV–vis light absorption of all the materials involved in PET depolymerization was measured. PET, EG, Pd‐CsPTA, and AC showed significant absorption in the UV range (Figure S18, Supporting Information), suggesting that the influence of UV lights needed to be examined.

**Figure 6:**
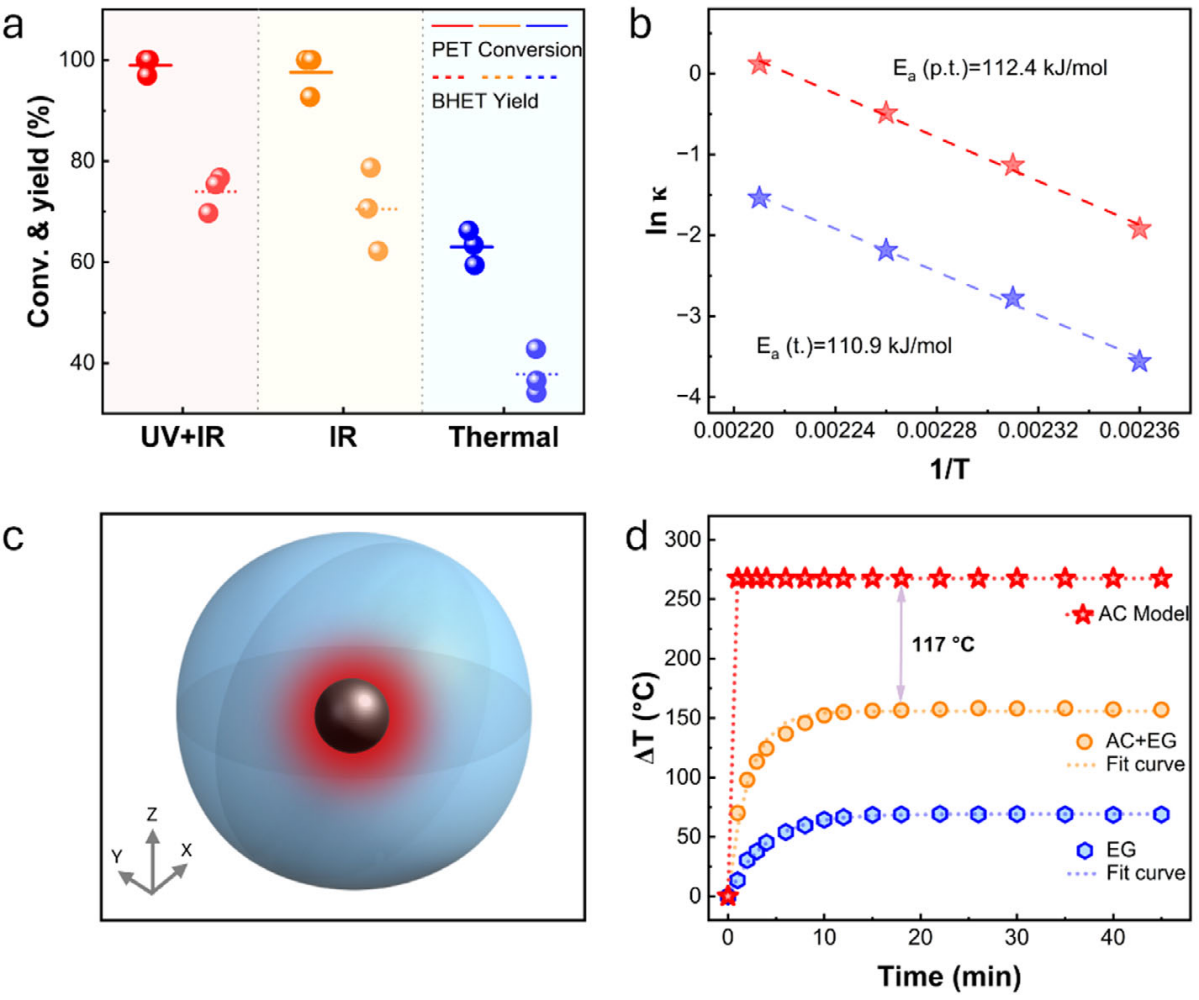
Localized heating effect. a) Effect of UV light in photothermal catalysis depolymerization of PET. b) Arrhenius plot of the rate constant of PET depolymerization in both photothermal (p.t.) and thermal (t.) cases. c) Microscale heat transfer model. d) The curves of temperature differences between different systems and the environment.

As shown in **Figure** **6**a, photothermal depolymerization experiments conducted in the absence of UV light resulted in a PET conversion rate close to 98% and BHET yield of 71%, similar to those achieved under full‐spectrum light irradiation. However, it was found that in the absence of UV wavelengths the heating rate and the maximum temperature declined (Figure S19, Supporting Information). Hence, the light power density had to be increased from 0.5 to 0.75 W cm^−2^ to achieve the desired temperature. The results suggested that the UV wavelengths contributed to photothermal conversion efficiency, but did not affect the depolymerization efficiency, which excluded the possibility of PET depolymerization through photocatalysis.

To further explore kinetic characteristics and mechanisms of the reaction, the rate constants (𝑘) and activation energies (*E*_a_) for photothermal and thermal (electricity‐powered) catalysis were calculated and compared. As shown in Figure S20 (Supporting Information), the data points for both reactions align closely along a straight line, indicating that there was a strong correlation between 𝑘 and temperature, with 𝑘 increasing as temperature rises. Across all the temperatures used, the 𝑘 values for photothermal catalytic reaction were consistently higher than those for the thermal catalytic reaction, suggesting that photothermal catalysis led to faster depolymerization. Moreover, the values of 𝑘 for photothermal catalysis exhibited a more significant response to temperature changes (Figure S20, Supporting Information), reflecting the greater sensitivity to temperature variations. *E*_a_ represents the energy barrier that reactants must overcome to transform into products.^[^ ^57^ ^]^ A linear fitting of ln𝑘 versus 1/T yielded a high correlation coefficient (*R* ^2^ close to 1), confirming the reliability of the calculated *E*_a_. The *E*_a._ for both reactions were nearly identical, indicating that light didn't significantly lower the energy barrier for the reactions (Figure 6b). Furthermore, the high level of *E*_a_ implied that bond breakage was the rate‐controlling step of the depolymerization process.^[^ ^57^ ^]^ Although *E*_a_ for photothermal catalysis and thermal catalysis are similar, the depolymerization efficiency of photothermal catalysis was markedly greater when the temperature of the bulk solution was the same. This difference may be caused by the localized heating effect of the AC particles, the surface temperature of which should be higher than the temperature of the bulk solution.^[^ ^38^ ^]^ During photothermal catalysis, AC absorbed light and converted it into heat, which diffused into the bulk solution. The higher surface temperature facilitated PET depolymerization. After 4 h of depolymerization at 180 °C, the depolymerization rate of thermal catalysis was lower than that of photothermal catalysis even at a lower temperature of 160 °C (Figure 5a). The reaction rate constant for thermal catalysis at 180 °C (0.216) was lower than that of photothermal catalysis at 160 °C (0.321) (Figure S19, Supporting Information).

To understand the localized heating effect, simulation was used to calculate the temperature discrepancies between the Pd‐CsPTA/AC surface and its surrounding environment. Compared to AC, the contribution of Pd‐CsPTA to photothermal conversion is negligible (Figure S21, Supporting Information). Hence, it was not considered in the simulation. The heat transfer from AC particles to the bulk solution is primarily through conduction. Hence, the following assumptions were made to establish the heat transfer model: 1) Photothermal materials (AC) absorbed light energy and transfered heat conductively to EG, leading to a temperature gradient within the system. 2) In non‐steady‐state heat transfer, the energy change rate of the system was represented by the difference between the heat generation and heat dissipation. 3) The model consisted of spherical AC particles, each with a diameter of 10 µm, encapsulated within an EG sphere (Figure 6c), the size of which was calculated based on the input ratio of AC to EG in the reaction system. The photothermal profile of the individual components, i.e., EG and AC particles were also calculated. As shown in Figure 6d, whether heating EG alone or heating the dispersing AC in EG, the temperature initially increased rapidly and then gradually stabilized. The temperature difference between the EG and the environment, as well as between the AC‐dispersed EG and the environment, were fitted using the least squares method. The fitted curves closely match the experimental data. Based on these fitted results, the temperature difference between the AC surface and the environment was calculated using the unsteady‐state heat transfer equation. The results indicated that at the steady state, the temperature difference between the AC surface and the temperature probe is 111.7 °C. This temperature difference indicated the existence of localized heating effect. The results from both experiments and calculations demonstrated that the chemical depolymerization pathway in photothermal catalysis was similar to the traditional thermal catalysis. However, the unique localized heating effect in photothermal catalysis could facilitate efficient depolymerization at lower overall reaction temperatures.

#### 2.4.2 Catalytic Mechanisms of Pd‐CsPTA

In the catalytic depolymerization of PET, Pd‐CsPTA exhibited excellent activity and stability, yet its precise catalytic mechanism remains unclear. Conventional characterization techniques provided valuable macroscopic insights but failed to capture the transient behavior of the catalyst and the formation of reaction intermediates. To address these limitations, in situ techniques were employed to monitor the structural changes of the catalyst and reactants under real reaction conditions.

**Figure 7:**
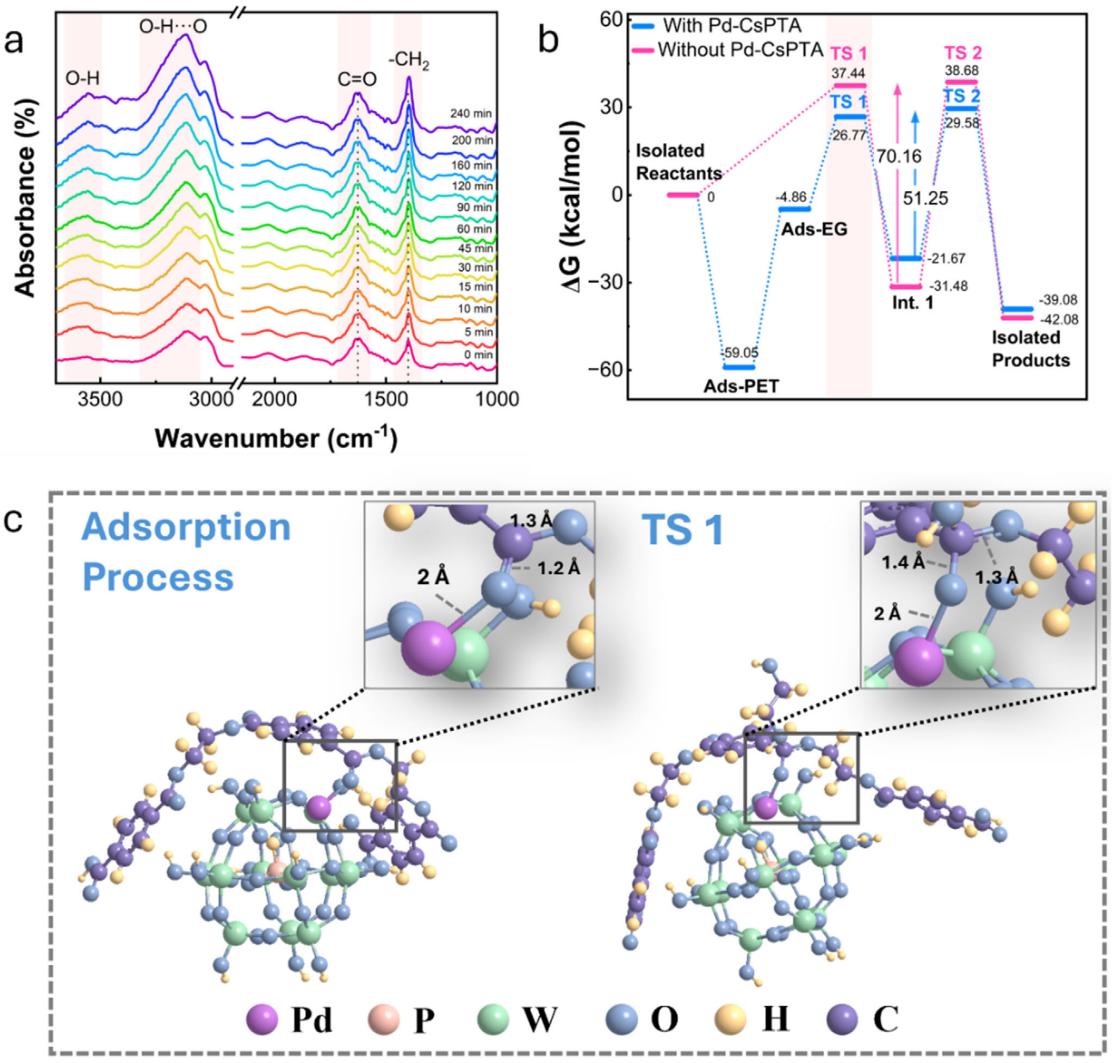
Catalytic mechanism. a) In situ FTIR measurement on PET depolymerization at 180 °C. b) Free energy distribution of Pd‐CsPTA catalyzed PET depolymerization through DFT. c) Molecular structure of PET and catalyst in adsorption state and transition state.

In situ infrared spectra revealed pivotal structural changes of PET during its depolymerization. As shown in **Figures** **7**a and S22 (Supporting Information), the characteristic C═O stretching vibration of PET shifted from 1710 to 1630 cm^−1^ at 0 min when the temperature reached 180 °C. This red shift indicates that coordination between Pd and the carbonyl oxygen had already occurred, which reduced the electron density of the C═O bond and led to a decrease in its vibrational frequency. The coordination bonds reduced the electron density in the carbonyl group, reducing the vibrational frequency of the C═O bond and causing its red shift. The characteristic peak at 3110 cm^−1^, corresponding to stretching vibration of hydrogen‐bonded hydroxyl (O──H···O), intensified progressively from the onset of the reaction (Figure S23a, Supporting Information), which indicated the involvement of more EG molecules to form intermolecular hydrogen bonds. Hydroxyl‐containing intermediates were continuously generated during the reaction to form hydrogen bonds with other atoms. Then the negatively charged hydroxyl oxygen in EG nucleophilically attacked the carbonyl carbon in PET, converting C═O to a single bond and forming a negatively charged intermediate. Subsequently, as the ester bond oxygen was leaved, an amount of monoester was released. Compared to PET, the C═O and ─CH_2_ in monoesters were more exposed and had greater vibrational freedom,^[^ ^58^ ^]^ leading to stronger absorption peaks (Figure S23b,c, Supporting Information).

To achieve an in‐depth understanding of the catalytic mechanism, DFT calculations were conducted to analyze the electronic distribution, interactions between the catalyst and reactant, and the key transition states and intermediates in the reaction pathway.^[^ ^59^ ^]^ In catalytic reactions, adsorption is the first step. By analyzing the charge distribution of PET, EG and Pd‐CsPTA, the possible adsorption sites can be identified. Subsequent comparison of adsorption energies helped to determine the most favorable adsorption site, thereby establishing the initiation point of the reaction. In PET, the carbonyl carbon was identified as the major positive charge center (Figure S24c, Supporting Information). In EG, hydroxyl oxygen held a significant negative charge (Figure S24b, Supporting Information), making it highly prone to nucleophilic attack on the carbonyl group of PET. The interaction between Pd and the carbonyl single‐bond oxygen in PET, as well as the weak π–π interaction with PET aromatic ring, were both relatively weak. However, Pd with an electron‐rich coordination environment showed a stronger preference for coordinating with the carbonyl oxygen of PET (Figure S25 and Table S5, Supporting Information and Figure 7c). This is consistent with the shift of the characteristic carbonyl peak observed in the in situ infrared spectra results. The formation of the coordination bond activated carbonyl carbon, further enhancing its positive charge, which facilitated the nucleophilic attack by the hydroxyl oxygen of EG (Figure S26b, Supporting Information). After the nucleophilic attack, the transition state (TS1) shown in Figure 7c and Figure S26c (Supporting Information) was formed. Next, the hydroxyl oxygen of EG formed a chemical bond with the carbonyl carbon, causing the carbon to undergo the transition from sp^2^ to sp^3^ hybridization. The coordination bond between the catalyst and PET was broken, leading to the formation of the intermediate (Int.1) (Figure S26d, Supporting Information).

By calculating the energy difference between the reactant and the transition state of the ester bond, the activation energy was determined to be about 26 kcal mol^−1^ (Figure 7b), close to the experimentally obtained value. The calculations indicated that the introduction of the catalyst significantly reduced this energy requirement by 10.67 kcal mol^−1^, enabling the reaction to proceed efficiently. The DFT results offered critical insights into the role of the catalyst in PET depolymerization, providing essential theoretical support for future experimental advancements and catalytic system design.

## 3 Conclusion

This work demonstrates the high catalytic efficiency of the phosphotungstic acid‐based palladium single‐atom catalyst (Pd‐CsPTA) supported on activated carbon (AC) particles, a material that can be obtained from cheap resources such as biomass, for the photothermal catalytic depolymerization of PET. The innovative catalyst design, combined with the strategic regulation of solar radiation direction, significantly improved both photothermal conversion efficiency and the depolymerization rate. Under simulated solar irradiation at an intensity of 0.5 W cm^−^^2^, the system rapidly reached 185 °C, enabling complete PET depolymerization within 4 h and achieving a 72% yield of BHET. The catalyst also demonstrated excellent recyclability with high PET conversion of 95% being maintained after five cycles. Mechanistic studies revealed that the electron‐rich local coordination environment around the Pd single‐atom sites facilitated strong interaction with the carbonyl oxygen of PET, thereby activating the carbonyl carbon and promoting the nucleophilic attack of ethylene glycol. This study presents an effective, sustainable and practically cost‐effective method for photothermal catalytic depolymerization of PET, advancing PET recycling technologies and opening new avenues for the design of highly efficient single atom‐catalysts.

## Conflict of Interest

The authors declare no conflict of interest.

## Supporting information



Supporting Information

## Data Availability

The data that support the findings of this study are available from the corresponding author upon reasonable request.
