# Supplementary material for: Highly Efficient Photothermal‐Catalytic Depolymerization of Polyester Fiber Enabled by a Phosphotungstate‐Based Palladium Single‐Atom Catalyst
Source: Small. 2025 Sep 15;21(44):e05673. doi: 10.1002/smll.202505673 (PMC12590518; doi:10.1002/smll.202505673)
Supplement: Supplementary file 1 — Supporting Information [file SMLL-21-e05673-s001.docx]

***Supporting information for:***

**Highly Efficient Photothermal-Catalytic Depolymerization of Polyester Fiber Enabled by a Phosphotungstate-Based Palladium Single-Atom Catalyst**

Xin Li,^1^ Yiming Bu,^1,2^ Lu Jiang,^1^ Yue You,^1^ Qi Han,^3^ Xiaokai Hu,^4, *^ Hongjun Yang,^5, *^ Joselito Macabuhay Razal,^1^ Christoper Hurren,^1^ Jingliang Li^1, *^

1. Institute for Frontier Materials, Deakin University, Waurn Ponds Campus, Geelong, Victoria 3216, Australia.

2. Future Industries Institute, UniSA STEM, University of South Australia, Mawson Lakes Campus, Adelaide, SA 5095, Australia.

3. School of Science, STEM College, RMIT University, Melbourne, VIC 3000, Australia

4. School of Mechanical and Electrical Engineering, Guilin University of Electronic Technology, Guilin 541004, China.

5. Key Laboratory for Green Processing & Application of New Textile Materials of the Education Ministry, Wuhan Textile University, Wuhan 430065, China.

**Methods**

**Chemicals and materials.** Polyethylene terephthalate (PET) fiber is a commercially available standard product. Powders of activate carbon (AC) were purchased from ACT Technologies PTY LTD. Phosphotungstic acid (PTA), palladium (II) nitrate hydrate (Pd (NO_3_)_2_), cesium nitrate (CsNO_3_) and ethylene glycol (EG) were purchased from Sigma-Aldrich.

**Synthesis of catalysts.** 216 mg of PTA was dissolved in 10 mL of deionized (DI) water and cooled in an ice bath to 0 ℃. CsNO_3_ (36 mg) and appropriate amount of Pd (NO_3_)_2_ were dissolved in 10 mL of DI water and added dropwise into the ice cooled PTA solution over 30 min. The mixture was stirred (500 rpm) for 5 h, centrifuged and washed 3 times, frozen for 24 h and then freeze-dried for 12 h. The method for preparing Pd-CsPTA/AC is similar to that for Pd-CsPTA, but with the addition of AC in the PTA. For the reference CsPTA materials, the procedure to synthesize the reference CsPTA material was similar to that of Pd-CsPTA but without Pd (NO_3_)_2_.

**Thermal and photothermal depolymerization of PET.** PET fiber (diameter= 50 μm) cut to 0.5 cm long. 0.2 g PET fibers were mixed with EG (3 g) and Pd-CsPTA (12 mg). For thermal depolymerization (without light irradiation), the mixtures were placed in a flask with a temperature monitor and mixed by magnetic stirring at 500 rpm. Then the reactor was heated on a hotplate for a required time. For photothermal depolymerization, Pd-CsPTA/AC (18 mg) was used instead of Pd-CsPTA. The mixture was placed in a quartz cell (700 µL, 2 mm light pass) and irradiated with simulated sunlight. To understand the influence of ultraviolet (UV) light on the depolymerization of PET, the UV band was removed using the instrument’s integrated filter.

**Collection of depolymerization products.** PET depolymerization productions were separated by hot filtration followed by recrystallization. At the end of reaction, the remaining PET solid was removed by filtration, washed with boiling water, dried and weighed. An appropriate amount of boiling water was added to the filtrate, which was stirred at 80 ℃ for 1 h, followed by vacuum filtration. After removing water, the second filtrate was placed in a refrigerator overnight to crystallize BHET.

The conversion of PET (C_PET_) and yield of BHET (Y_BHET_) were calculated by the following equations, respectively:

(Eq. S1)

$$C_{PET}=\frac{{(W}_{0}-W_{t})}{W_{0}} \times100\%$$

(Eq. S2)

$$Y_{BHET}=\frac{W_{BHET}/MW_{BHET}}{W_{PET}/MW_{PET}} \times100\%$$

where $W_{0}$ and $W_{t}$ (g) represent the initial and residual mass of PET, respectively; $W_{BHET}$, $W_{PET}$, $MW_{PET}$, and $MW_{BHET}$ (g) correspond to the mass of BHET, initial weight of PET, molecular weight of PET repeating unit, and molecular weight (g/mol) of BHET, respectively.

The spatio-temporal yield (STY) was calculated as follows:

$STY=\frac{mass of product (g)}{reaction volume\left( L \right)*total reaction time (h)}$ (Eq. S3)

**Materials Characterization.** TEM images and relative EDS element distribution was collected by a JEOL 2100 LAB6 and FEG JEOL 2100F at 200 KV. SEM images were obtained by a Zeiss Supra 55 VP SEM. Powder X-ray diffraction was performed using Powder Panalytical X-Pert. X-ray absorption fine structure (XAFS) spectroscopy was carried out at 4-channel Silicon Drift Detector Bruker 5040 to investigate the local coordination environment and chemical state of the metal atoms. Data analyse were conducted using Athena and Artemis included in the Ifeffit and Demeter package. Fourier Transform Infrared Spectroscopy (FTIR) was used Bruker, Invenio-R. In situ infrared spectroscopy (Thermo iS50 FTIR Spectrometer) was used for real-time monitoring of chemical bond changes during PET depolymerization. Raman spectra were recorded using the InVia Raman Microscope with a wavelength of 514 nm. XPS spectra (Kratos AXIS Supra XPS) was used to analyze elemental composition and determine chemical state. Thermogravimetric Analysis was using Discovery TGA 550 to assess the thermal stability of the catalyst. The specific surface area and pore size distribution of the material were measured using Quantachrome, Autosorb iQ instruments. UV-visible absorption spectra were recorded by Spectrometer UV /Vis/NIR-DRA (Carry 5000) and UV/Vis-Liquid (Cary, 300) in transmission mode. High Performance Liquid Chromatography-Mass Spectrometry, Water was used to test products. All solar experiments were conducted by solar simulator (CEL-S500-t5). The temperature of the reaction system was monitored by a AS887 multi-channel sensor. The irradiated surface temperature of the reactor was measured by an infrared thermal camera (FLIR T420).

**Heat conduction simulation.**

The process in which AC converted light energy into heat energy and then transferred it to EG was defined using an unsteady-state heat conduction model, where the internal energy change rate was represented by the difference between the heat generation and heat dissipation powers, as shown in the following equation.

(Eq. S4)

$$\rho C\frac{d(\Delta T)}{dt}=Q-H\cdot\Delta T$$

where ρ (g/cm^3^) and *C* (J/K/cm^3^) represent the density and specific heat of the substance; *ΔT* (℃) and *t* (℃) correspond to temperature change and time; *Q* (J/min/cm^3^) and *H* (J/(K⸱cm^3^⸱min) are the heating power and heat dissipation coefficient.

Furthermore, this model involved AC and EG, and it was governed by the following three heat transfer differential equations and two relations.

(Eq. S5)

$${{(r}_{1}C}_{1}\rho_{1}+r_{2}C_{2}\rho_{2})\frac{d(\Delta T)}{dt}=Q_{t}-H_{t}\cdot\Delta T$$

(Eq. S6)

$$C_{1}\rho_{1}\cdot\frac{d(\Delta T)}{dt}=Q_{1}-H_{1}\cdot\Delta T$$

(Eq. S7)

$$C_{2}\rho_{2}\cdot\frac{d(\Delta T)}{dt}=Q_{2}-H_{2}\cdot\Delta T$$

(Eq. S8)

$$Q_{t}=Q_{1}r_{1}+Q_{2}r_{2}$$

(Eq. S9)

$$H_{t}=H_{1}r_{1}+H_{2}r_{2}$$

where *r* represents the volume fraction. The physical quantities with subscript *t* relate to the properties of the overall model, where AC was wrapped by EG and the letters with subscripts *1* and *2* refer to the physical quantities associated with AC and EG respectively.

Before fitting the experimental data, the heat transfer equation was simplified to Eq. S12 through the definitions of *q* and *τ*.

(Eq. S10)

$$q=\frac{Q}{\rho C}$$

(Eq. S11)

$$\tau=\frac{\rho C}{H}$$

(Eq. S12)

$$\frac{d(\Delta T)}{dt}=q-\frac{\Delta T}{\tau}$$

The temperature rise curves of the EG-encapsulated AC model and EG are fitted using the least squares method to obtain the intermediate variables q_t_, *τ* _t_, q_2_, and *τ* _2_, which provide the best fit of the theoretical model to the experimental data. Finally, the surface temperature variation of AC over time can be determined using Eq. S10, S11, S8, S9 and S5.

**DFT calculations.** All of the free energies of the reactants were computed using DFT in conjunction with the Gaussian 16 software package.^[1]^ The geometry of the Pd-CsPTA model was optimized with the PBE0 functional,^[2]^ incorporating the DFT-D3 dispersion correction with BJ damping ^[3]^ and the def2-SVP basis set. Then, all relevant spin states of PET and Pd-CsPTA in the reactant, transition state, and product regions were evaluated, including both low-spin and high-spin states. The lowest free energy spin multiplicity was chosen to calculate the thermodynamic properties (ΔG) and free energy barrier (ΔG‡) for the PET depolymerization process. To improve the accuracy of the free energy values, single-point calculations were performed at all stationary points with the same functional and a larger def2-TZVP basis set.^[4, 5]^ Additionally, the SMD implicit solvation model ^[6]^ was employed to account for the solvation effects of EG.


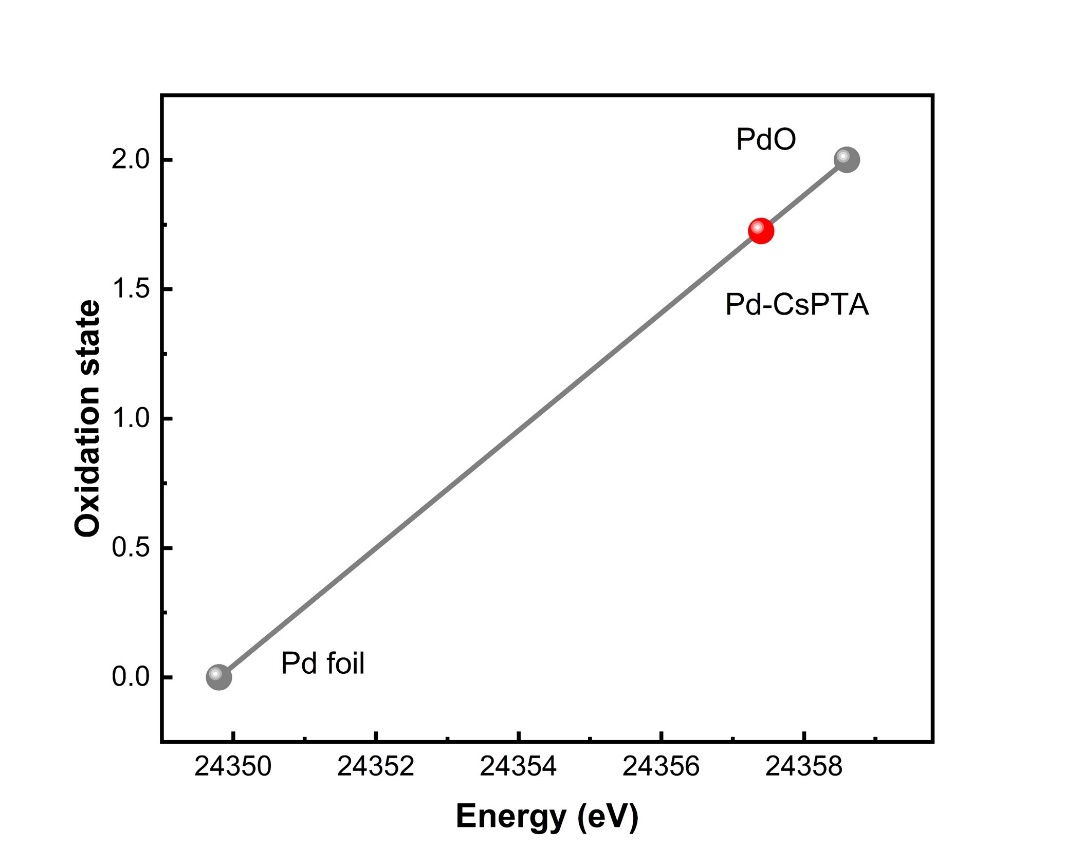


**Figure S1**. Linear fitting curve of oxidation states derived from corresponding Pd k-edge XANES spectra of Pd-based samples.


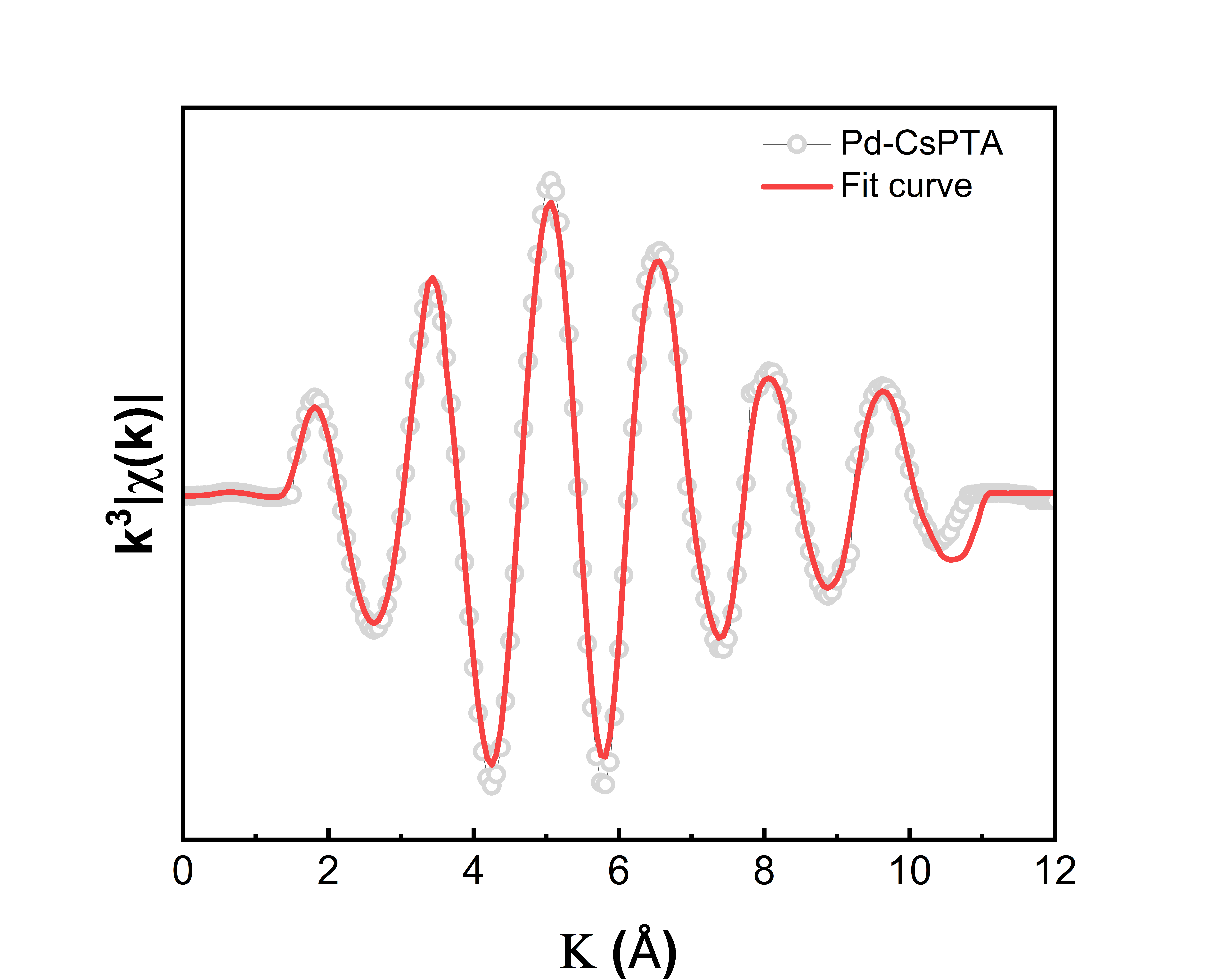


**Figure S2**. K-space fitting EXAFS of Pd-CsPTA.


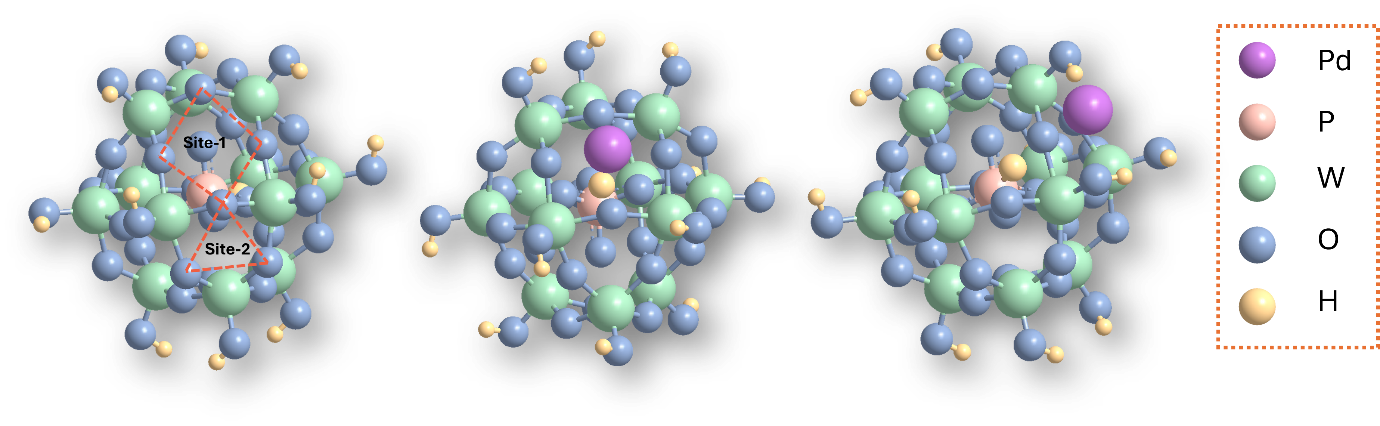


**Figure S3**. Molecular structure of Pd coordinated on PTA.


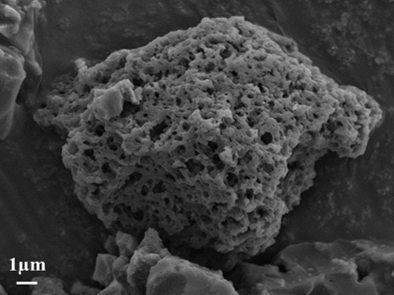

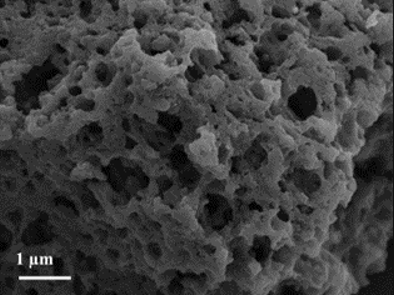


**Figure S4**. SEM images of AC particles.


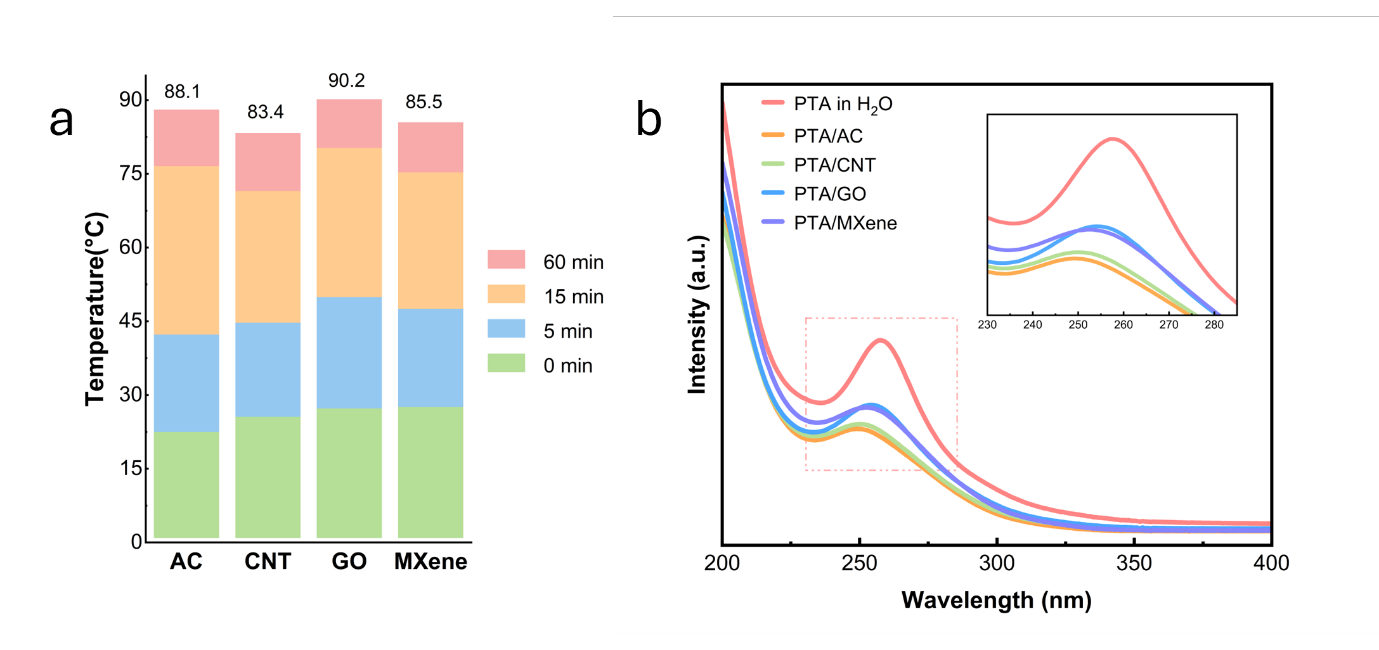


**Figure S5**. a) Photothermal properties and b) PTA adsorption properties of common photothermal materials (AC, carbon nanotube (CNT), graphene oxide (GO) and MXene).


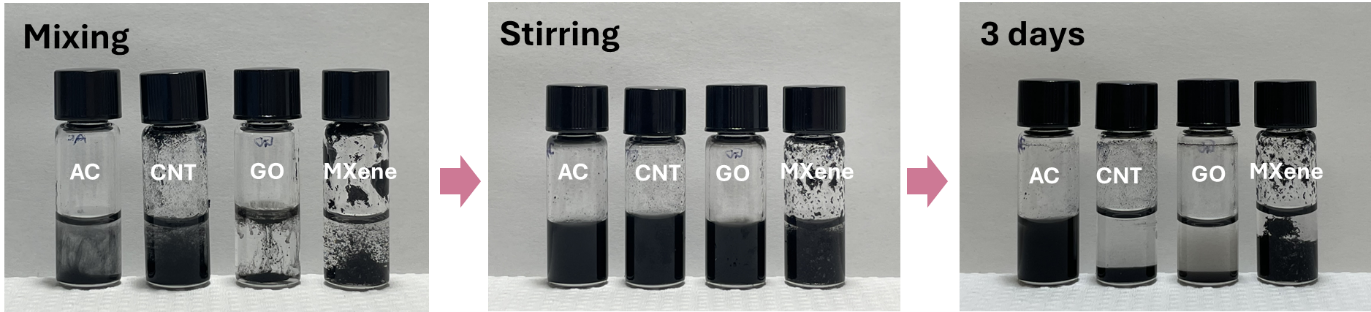


**Figure S6**. Dispersion of AC, CNT, GO and MXene in EG. AC particles showed superior stability in EG.


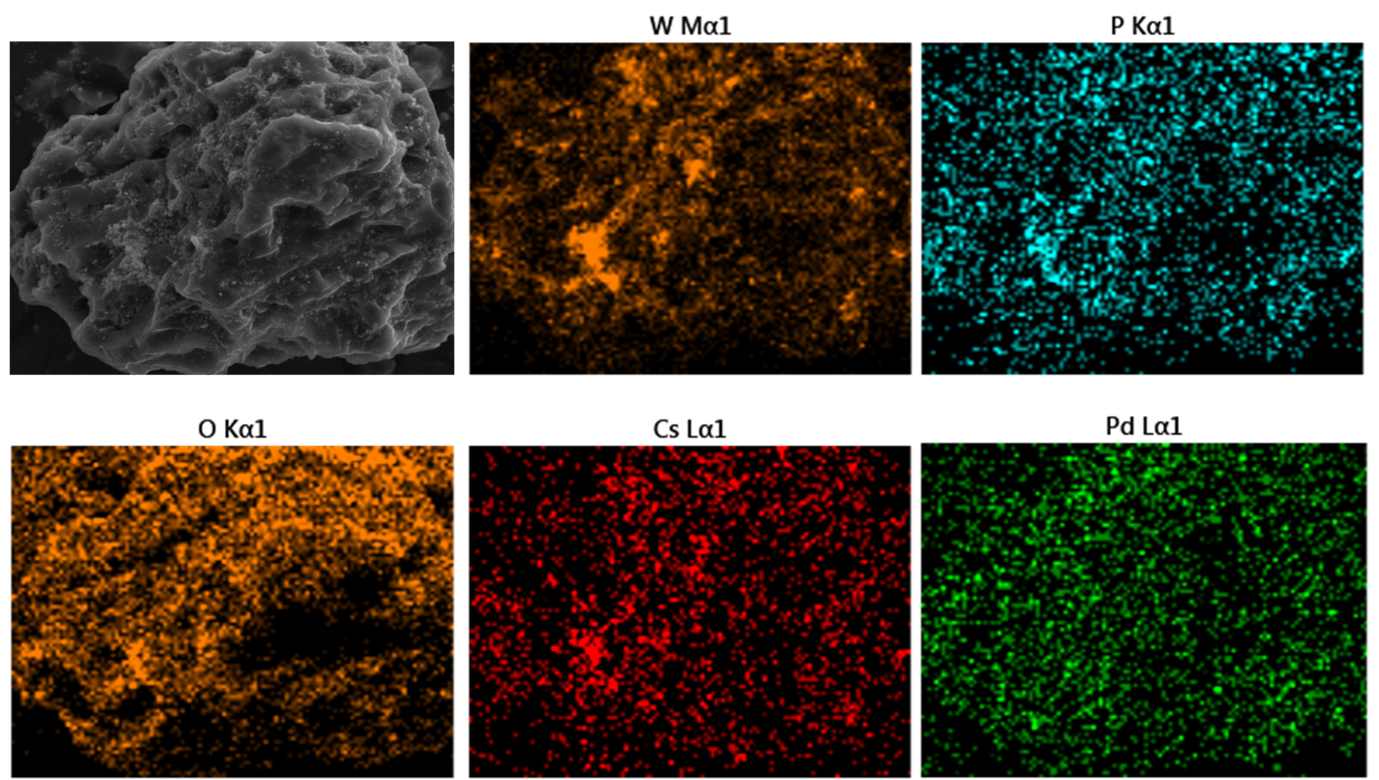


**Figure S7**. EDS images of Pd-CsPTA/AC.


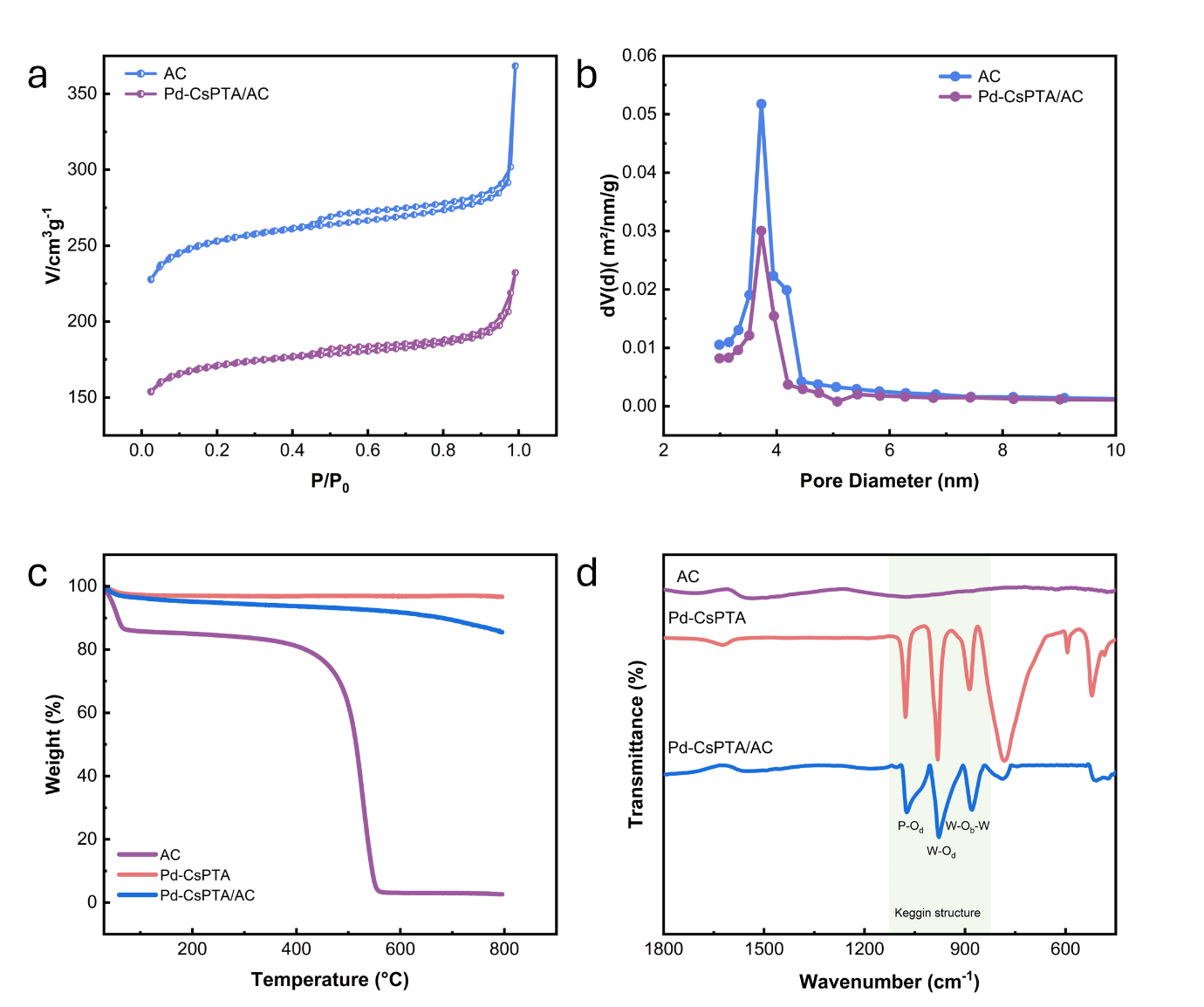


**Figure S8**. Characterizations of catalysts. a) N_2_ adsorption and analysis curves and b) pore size distribution curves of AC and Pd-CsPTA/AC. c) thermogravimetric analysis and d) FTIR of AC, Pd-CsPTA and Pd-CsPTA/AC.


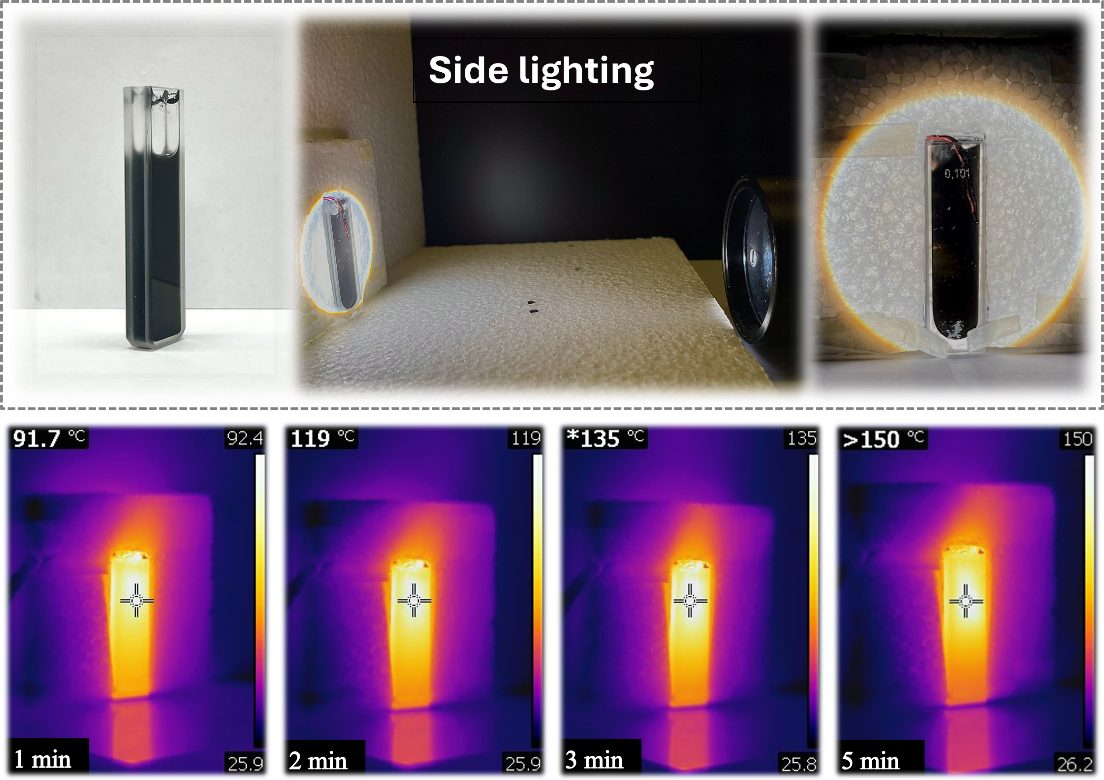


**Figure S9**. Photothermal catalytic depolymerization device (2 mm light pass, 700 µL), and temperature of the system under different illumination times.





**Figure S10**. Comparison of reported photothermal systems. Stability is the number of heating-cooling cycles. PTM is photothermal materials.


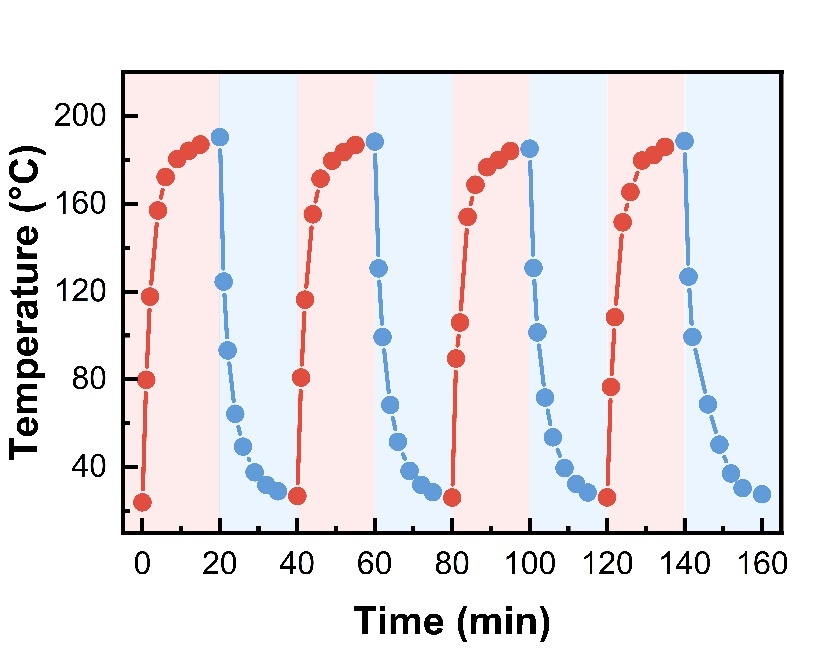


**Figure S11**. The heating-cooling cycles of Pd-CsPTA/AC in EG.


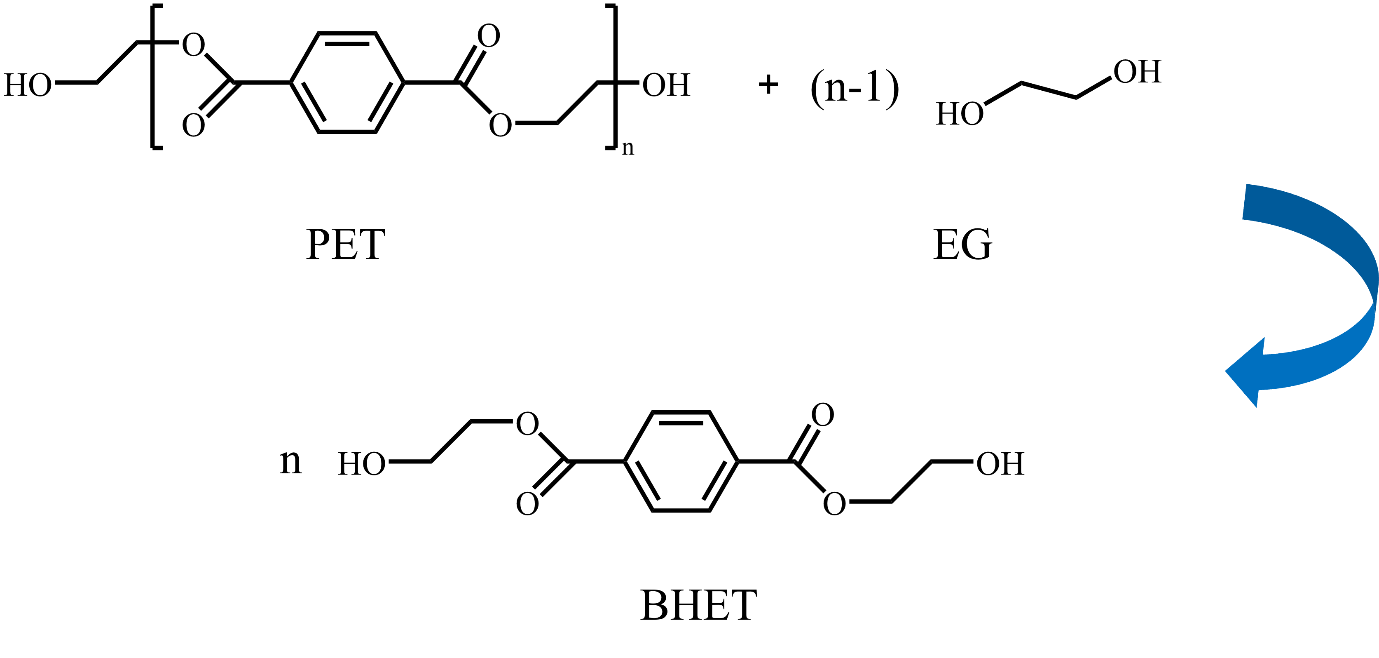


**Figure S12**. Schematic illustration of PET depolymerization through glycolysis.


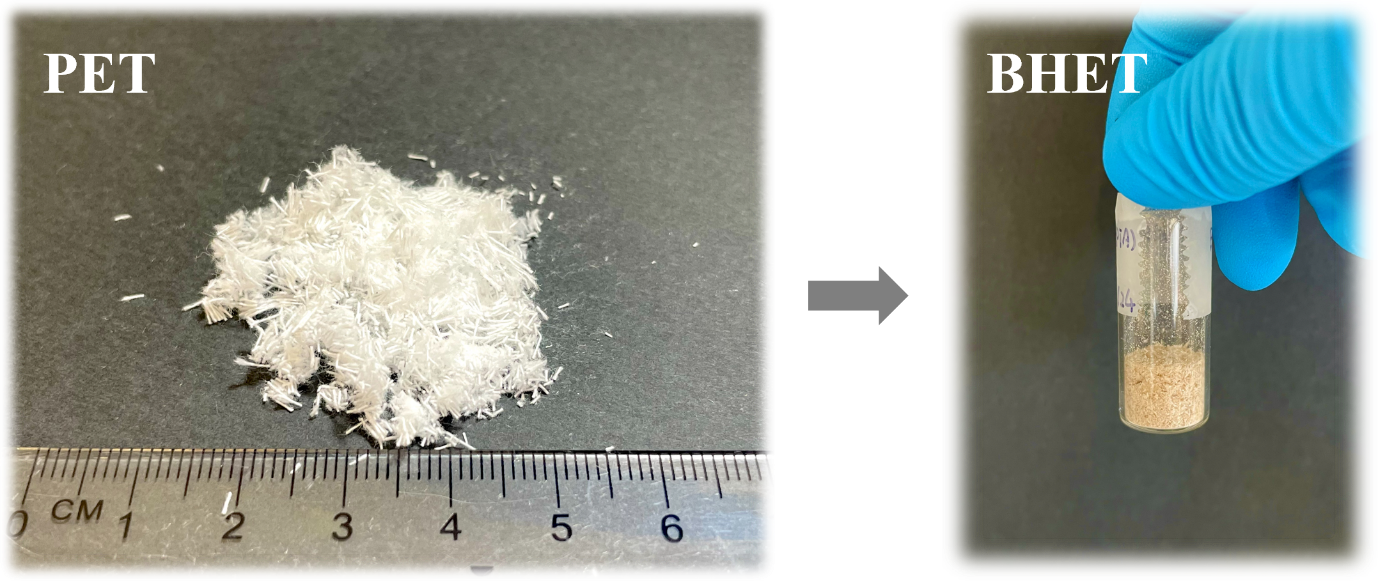


**Figure S13**. Digital images of PET fibers and recycled BHET crystals.


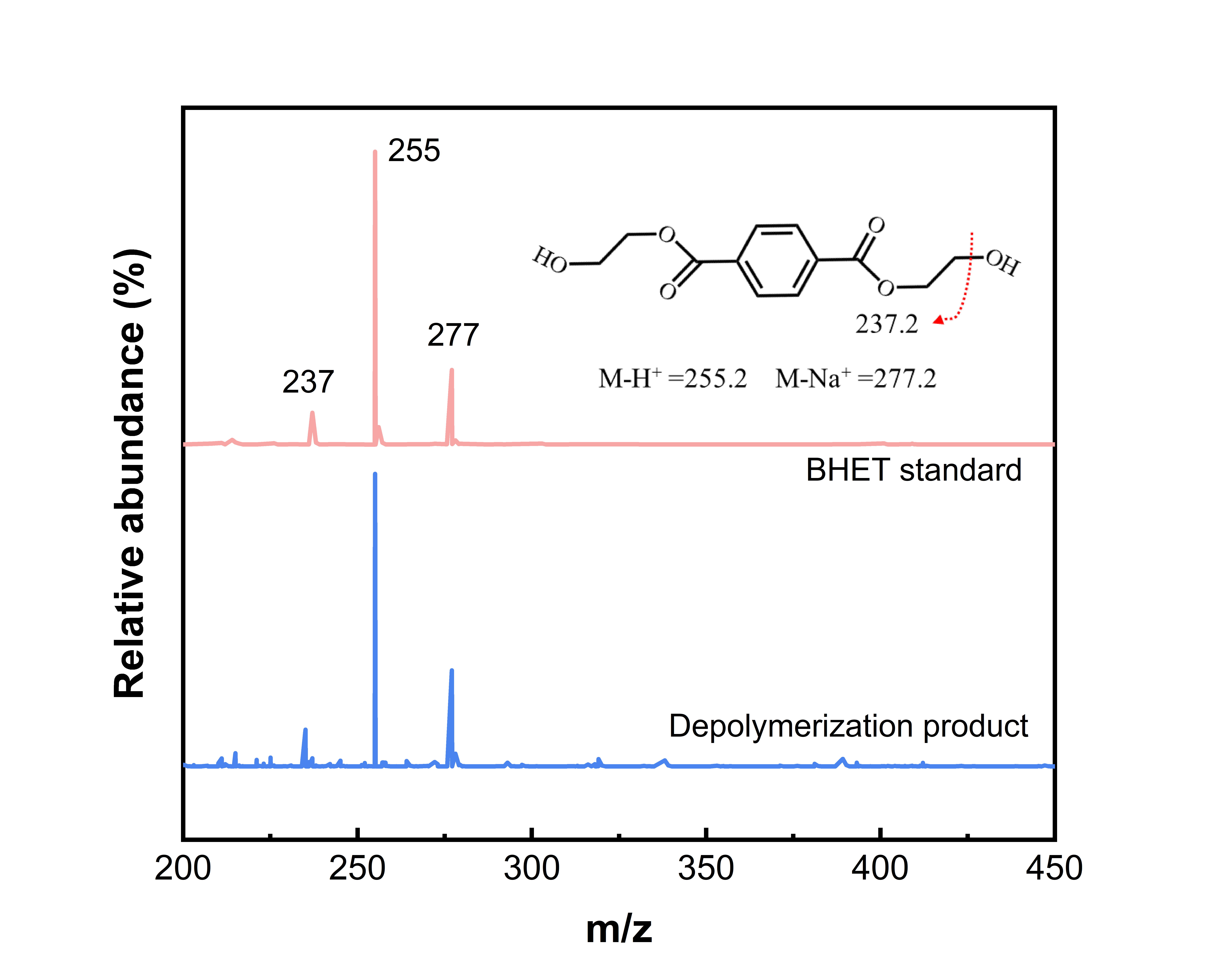


**Figure S14**. LC-MS/MS spectrum of the depolymerization product and BHET standard.


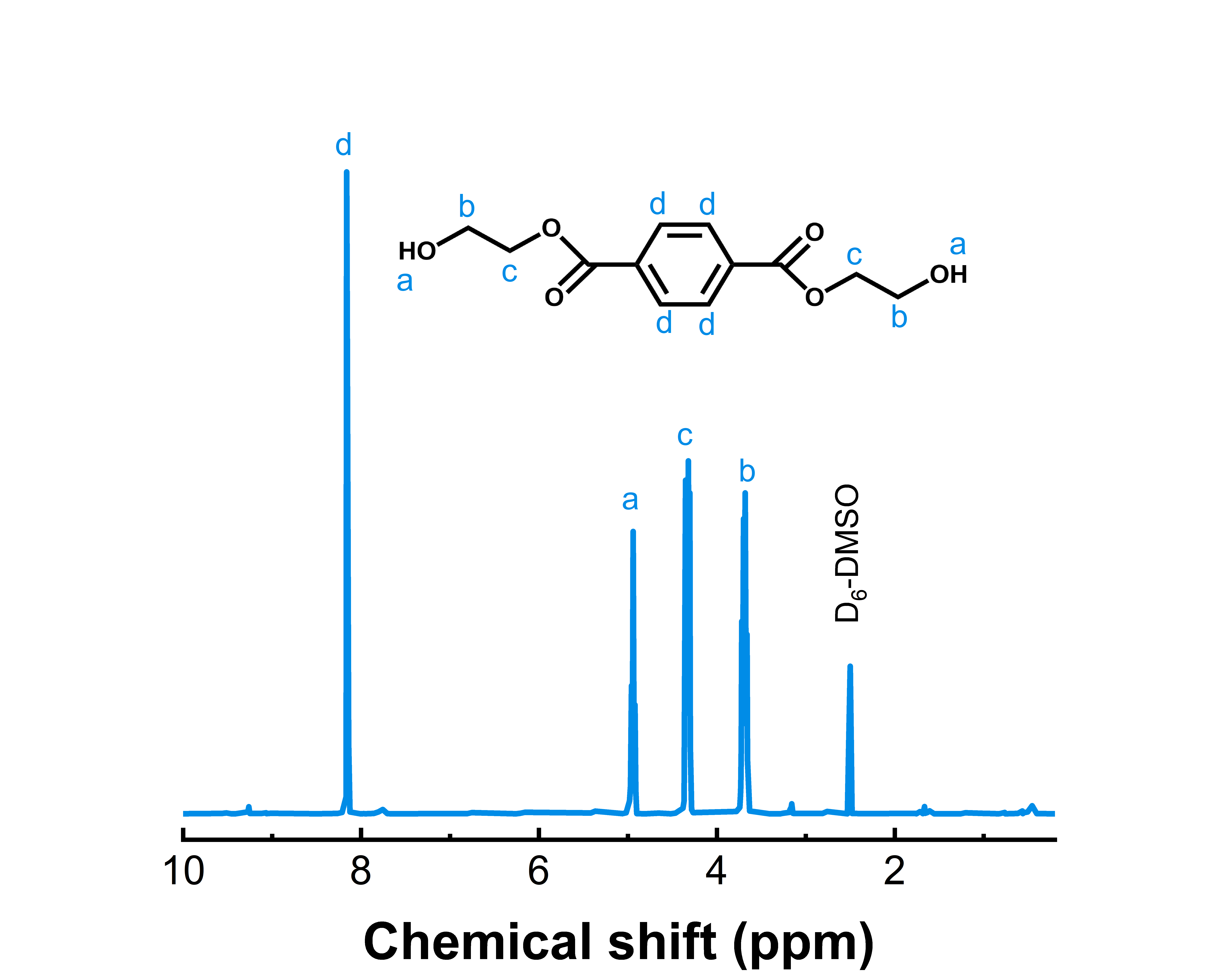


**Figure S15** ^1^H NMR spectra of depolymerization product.


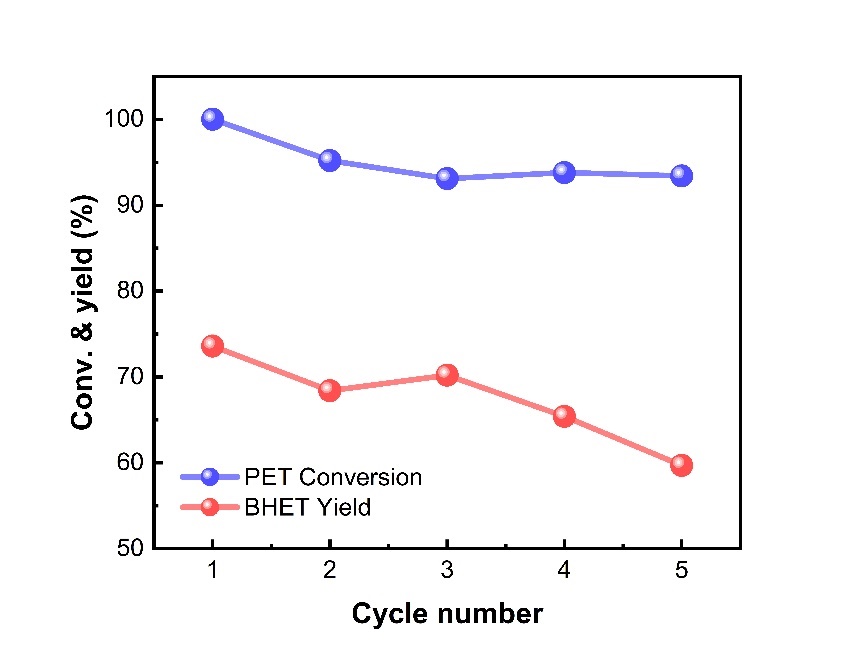


**Figure S16**. Catalytic performance of Pd-CsPTA during repeated uses.


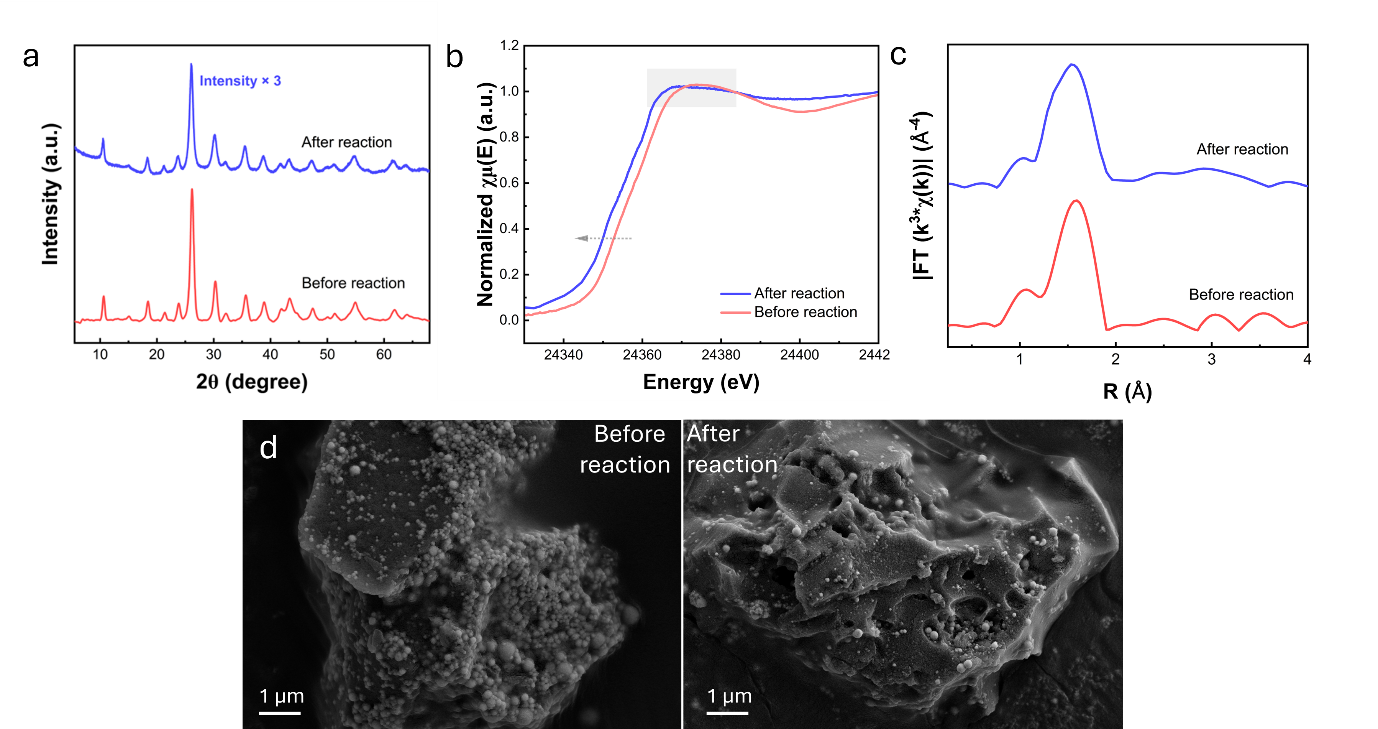


**Figure S17.** XRD pattern, normalized XANES, K^3^-weight FT-EXAFS spectra of and SEM images of catalysts before and after reaction (recycled 5 times).


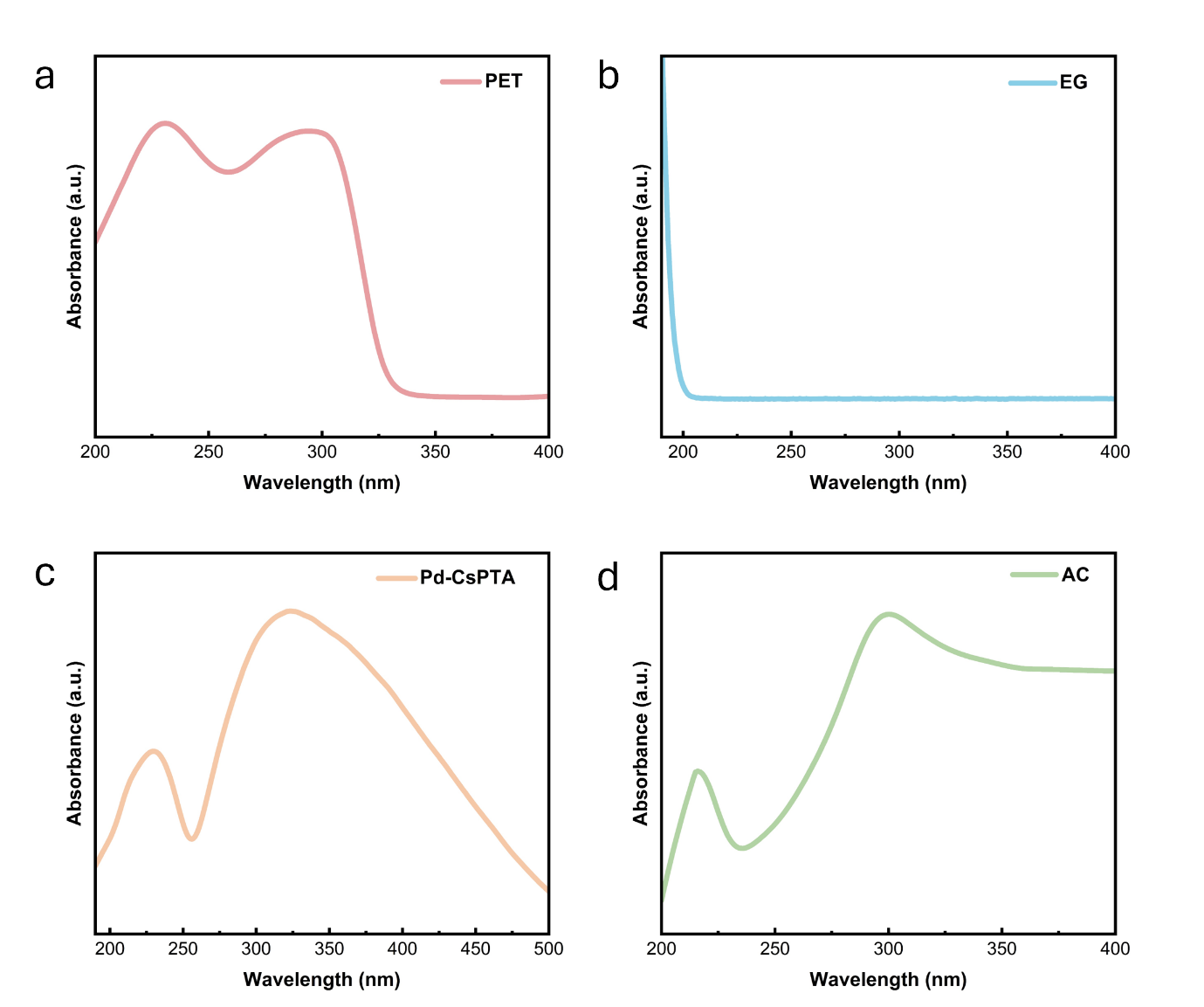


**Figure S18**. UV-vis spectra of a) PET, b) EG, c) Pd-CsPTA and d) AC.


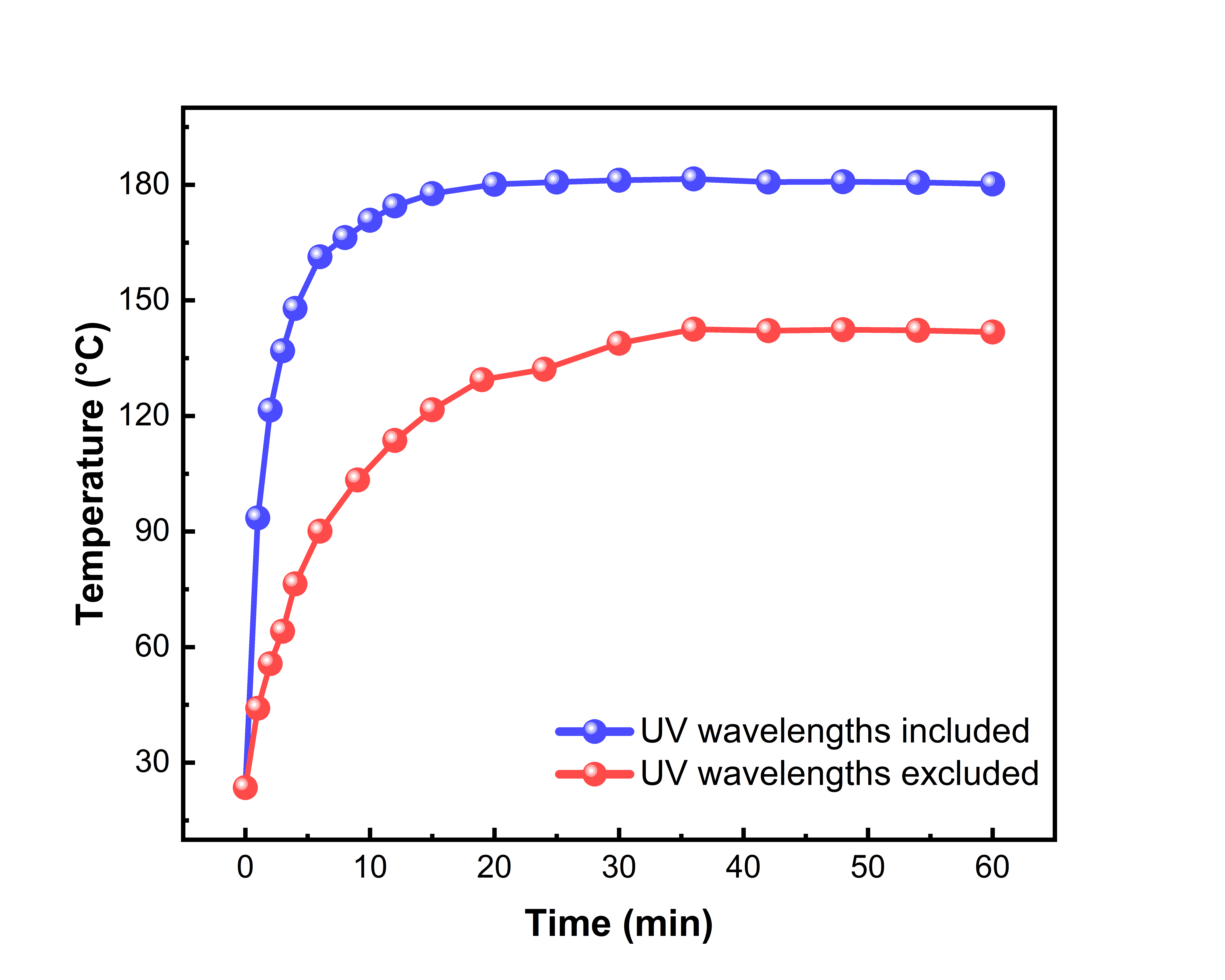


**Figure S19**. Temperature of the photothermal system over time under different sunlight wavelength.


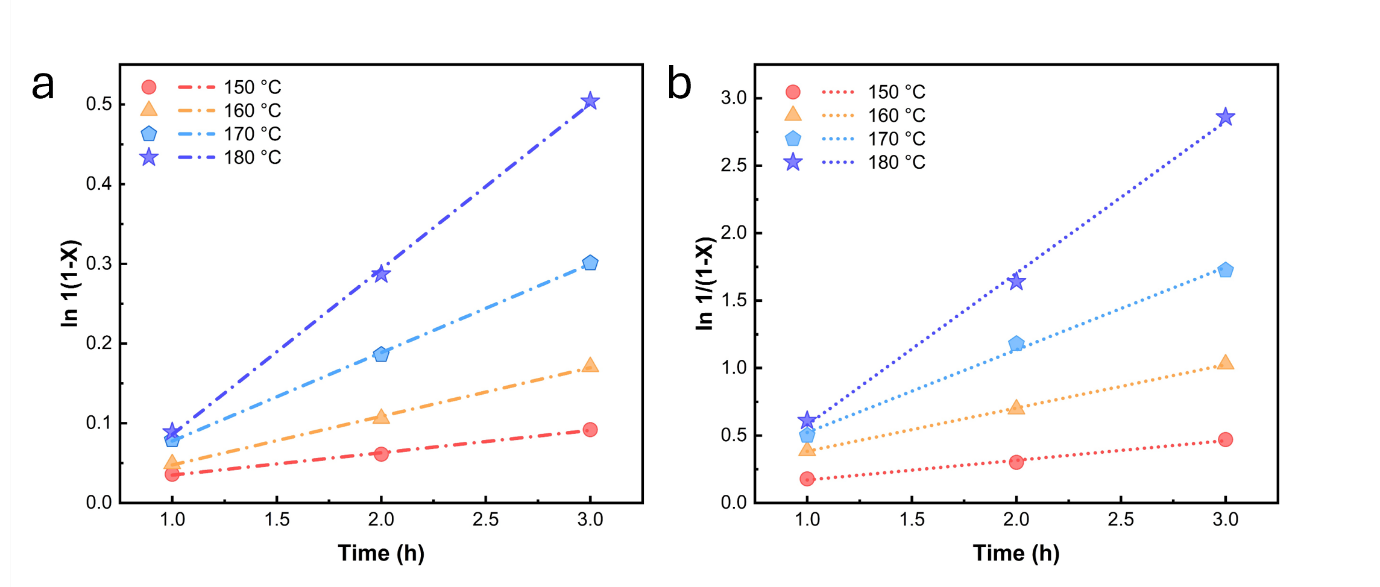


**Figure S20**. Effect of the temperature on the rate of PET depolymerization in a) thermal catalysis and b) photothermal catalysis.


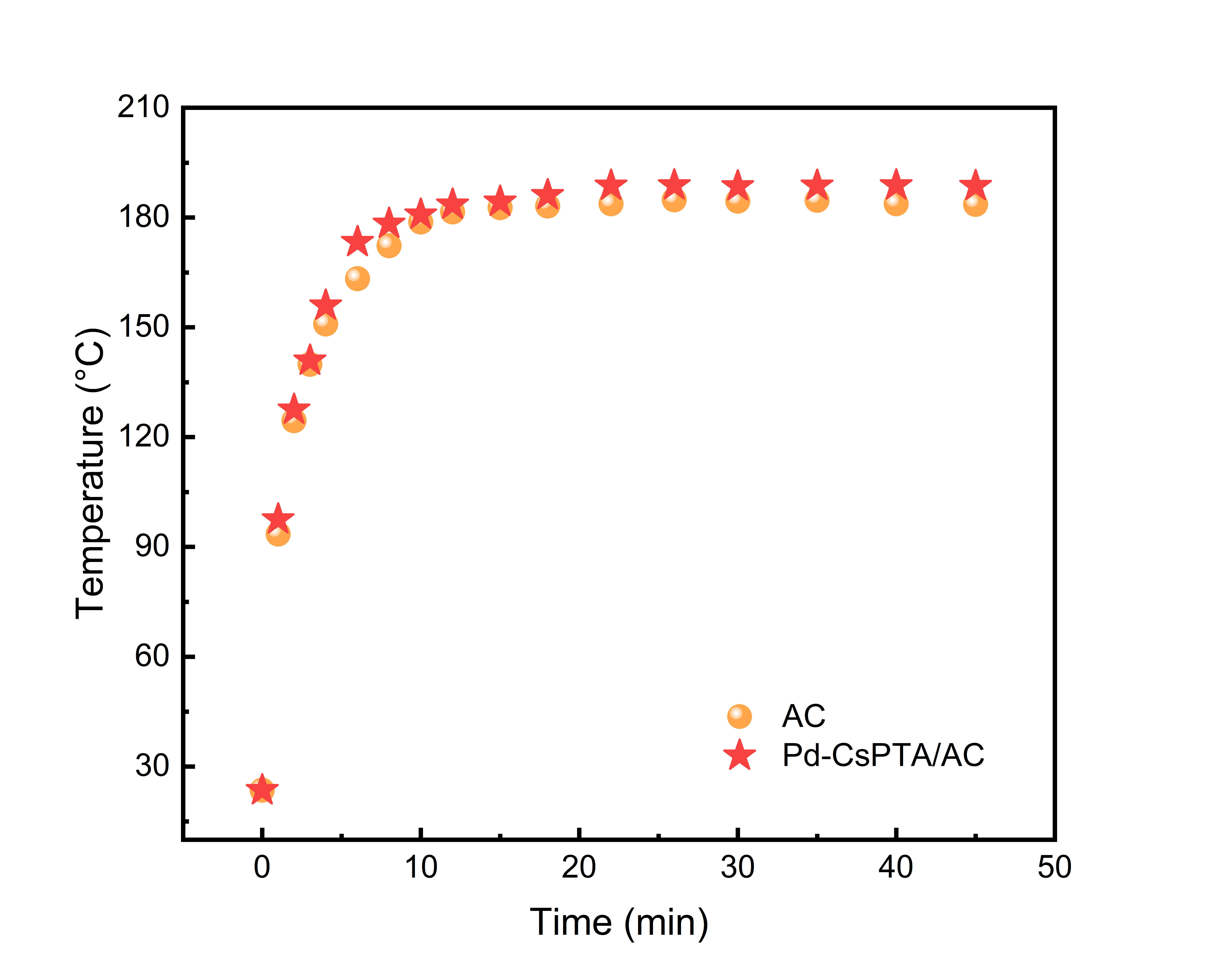


**Figure S21**. Temperature changes of AC and Pd-CsPTA/AC in EG.


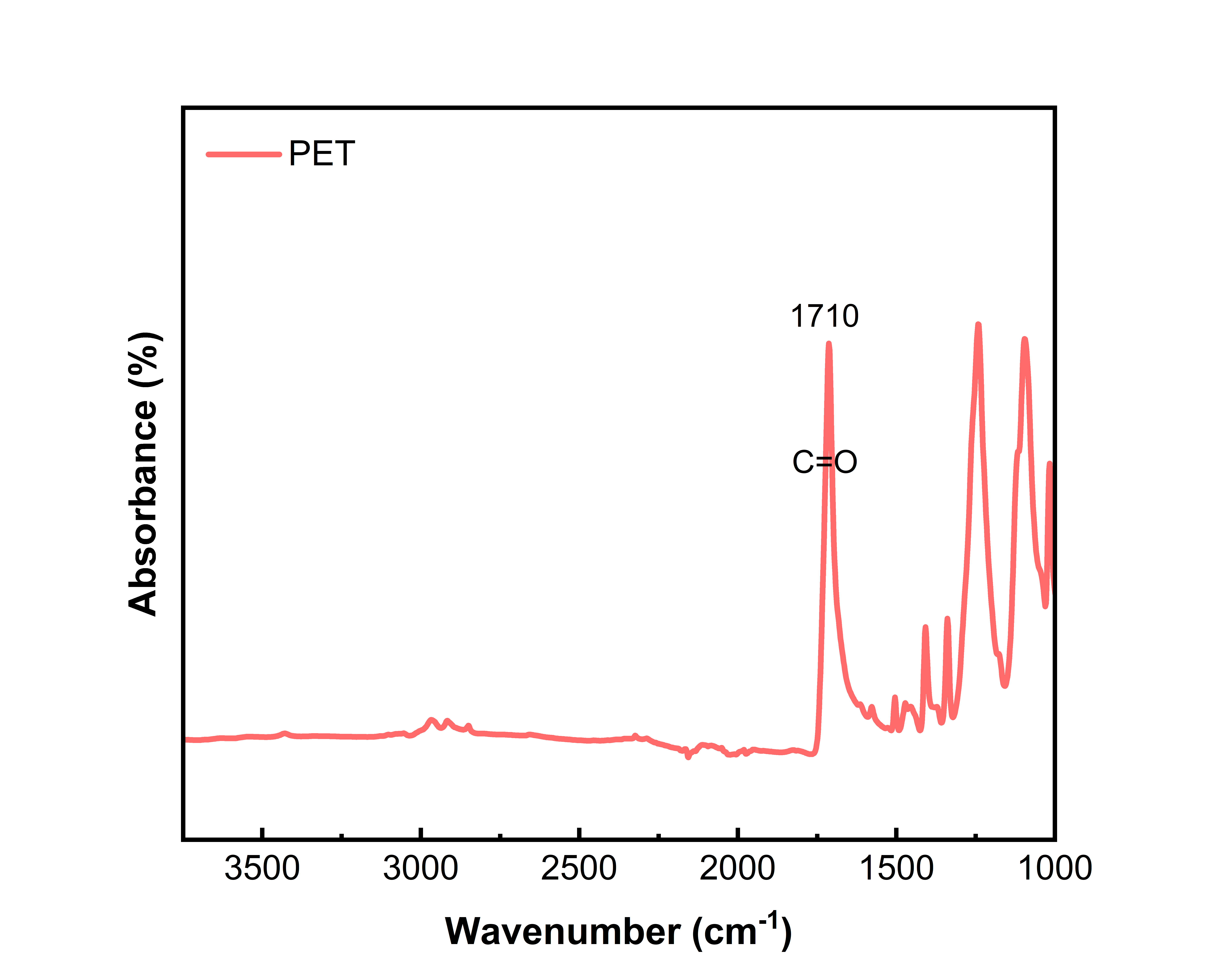


**Figure S22**. FTIR spectrum of PET.


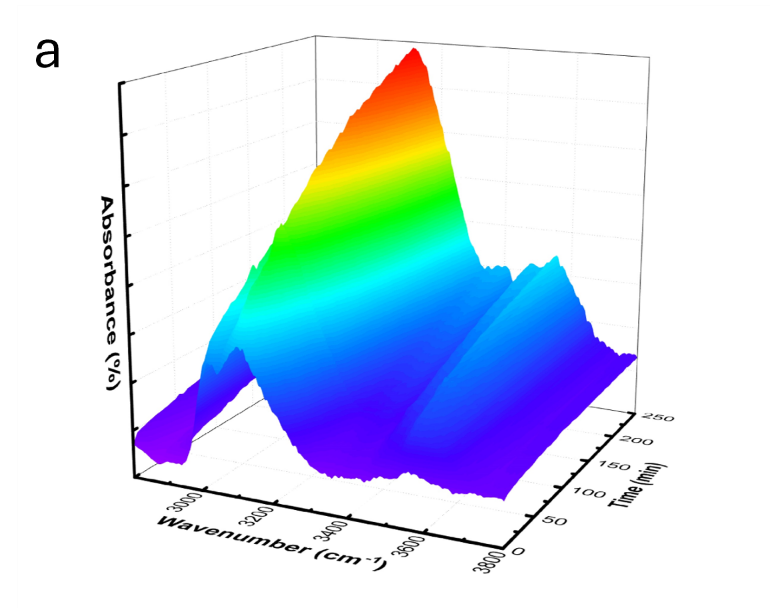


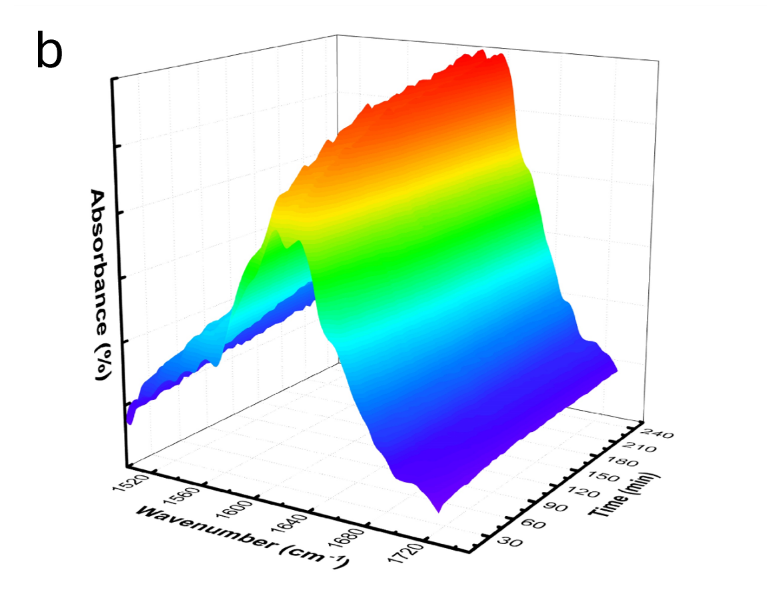


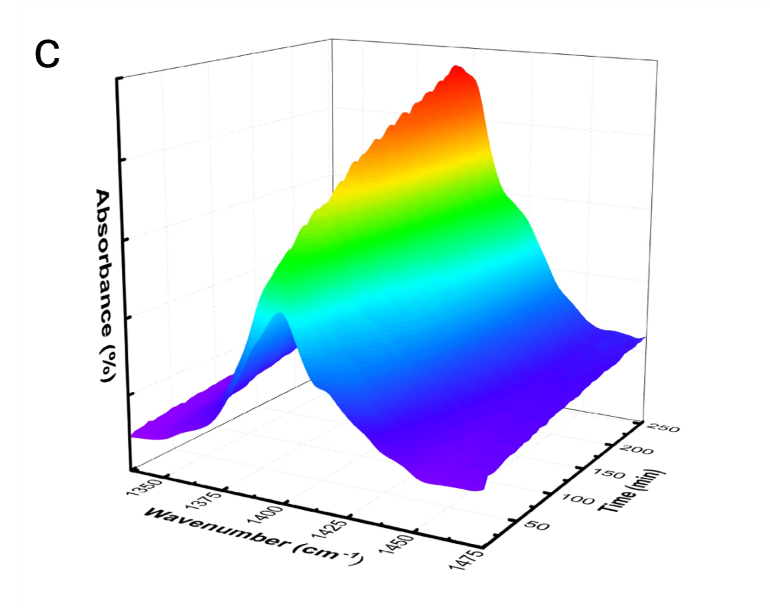


**Figure S23**. Curves of in-situ FTIR characteristic peak intensity changes with time. a) 2800-3600 cm^-1^, b) 1520-1720 cm^-1^ and c) 1360-1475 cm^-1^.


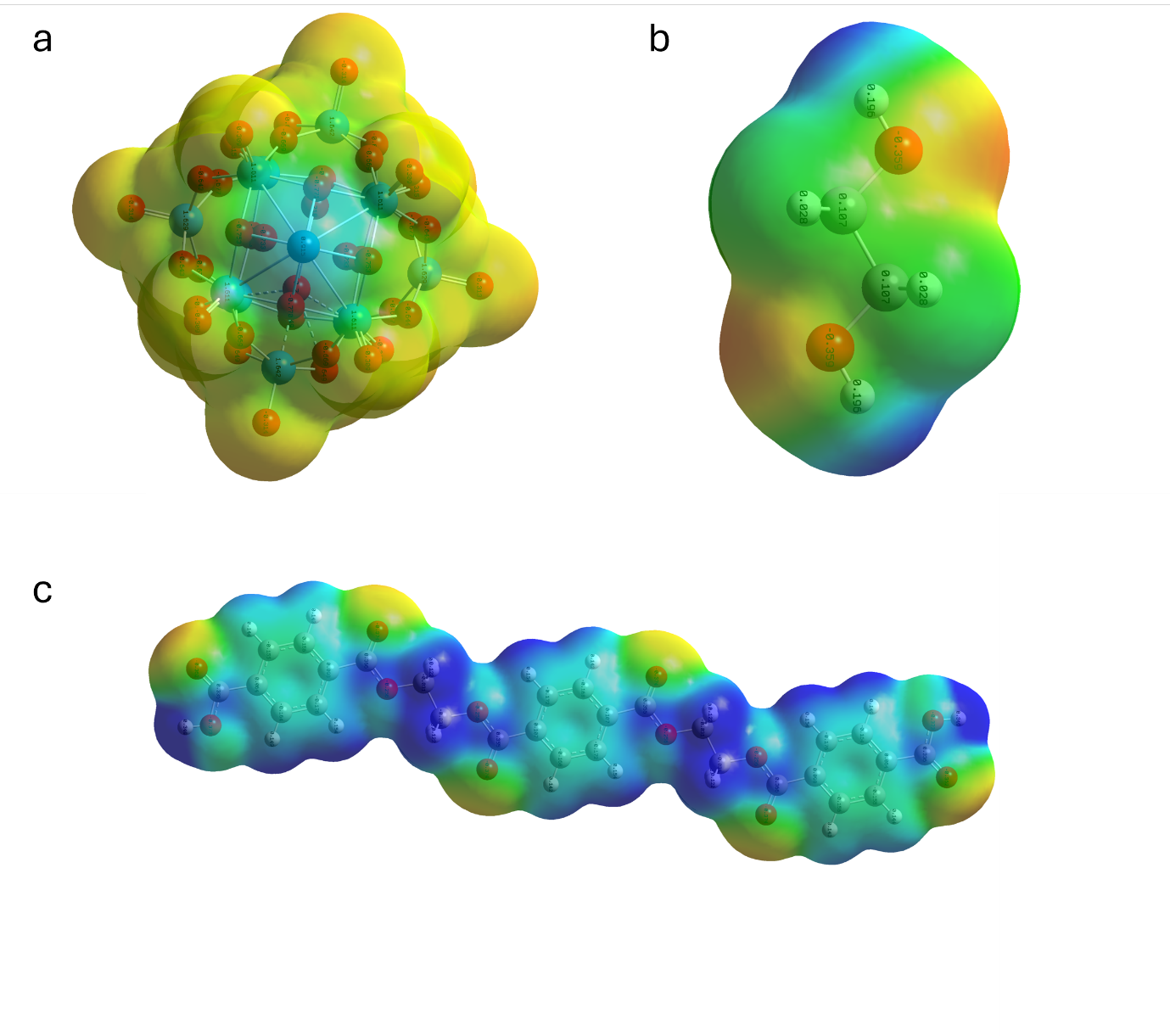


**Figure S24**. Molecular electrostatic potential images of a) Pd-CsPTA, b) EG and c) PET.


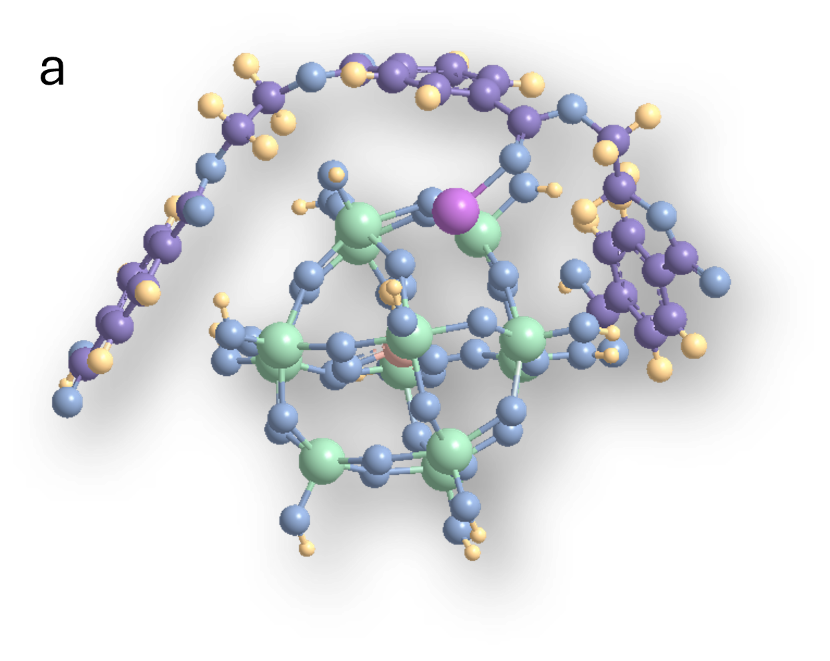


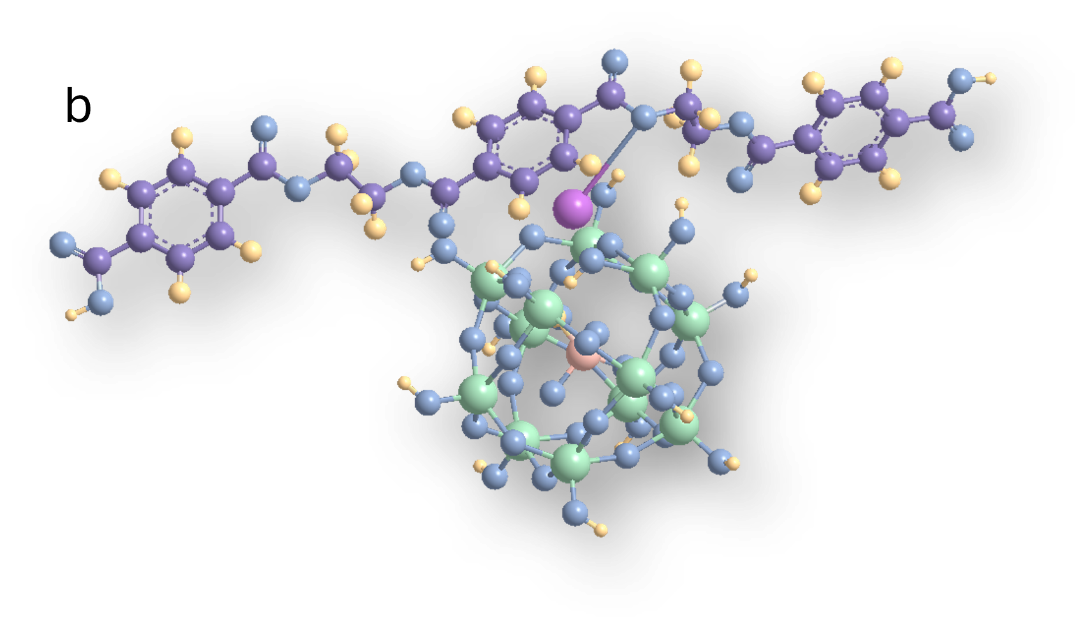


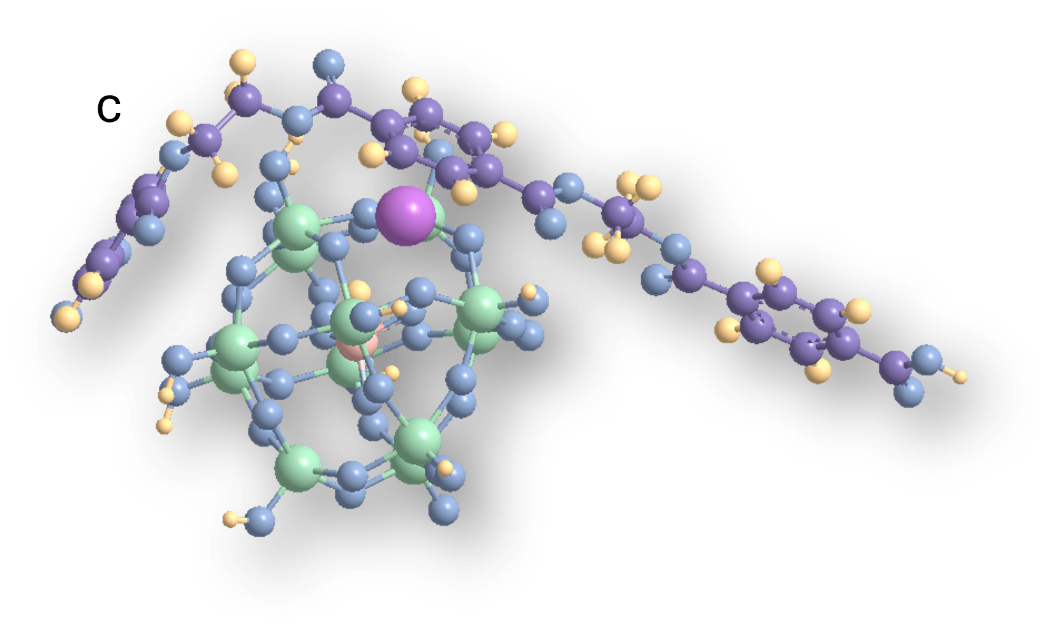


**Figure S25**. Schematic diagram of the interaction between PET and Pd-CsPTA/AC. Pd coordinated with the a) double and b) single bond oxygen in the carbonyl group of PET. c) The weak π-π interaction with PET’s aromatic ring.


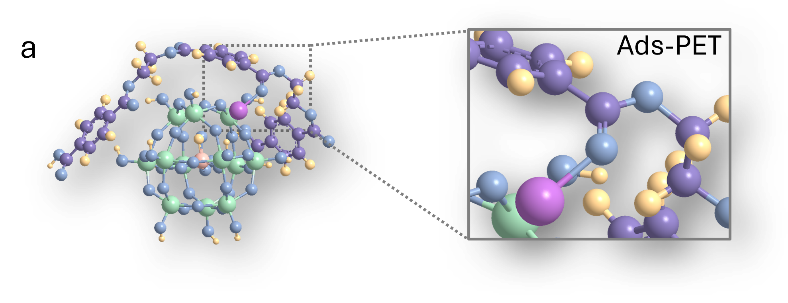


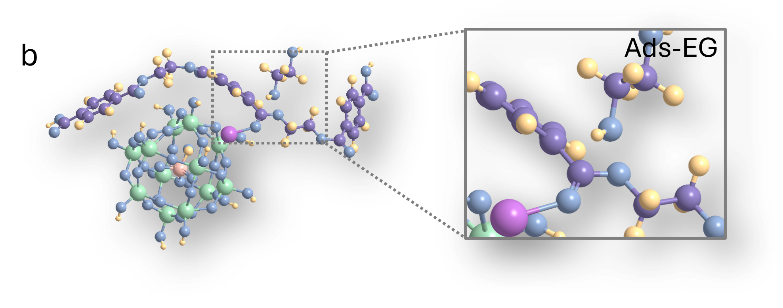


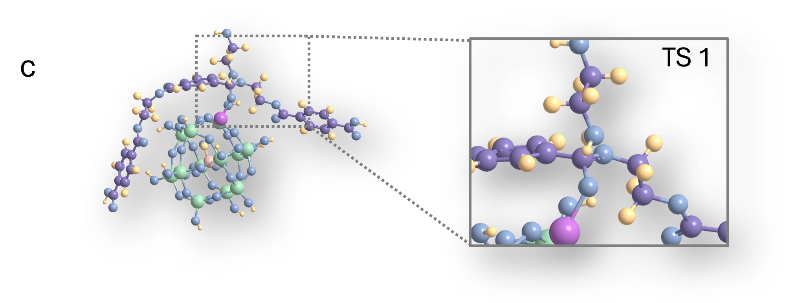


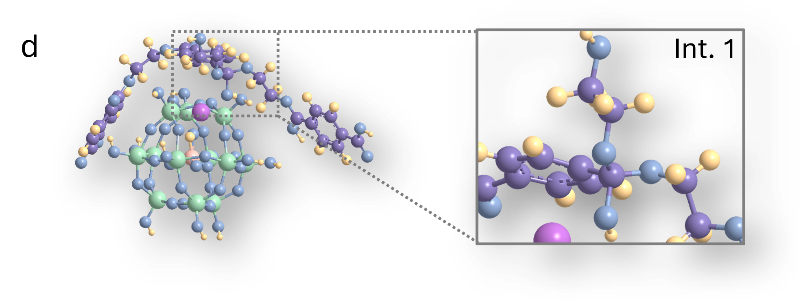


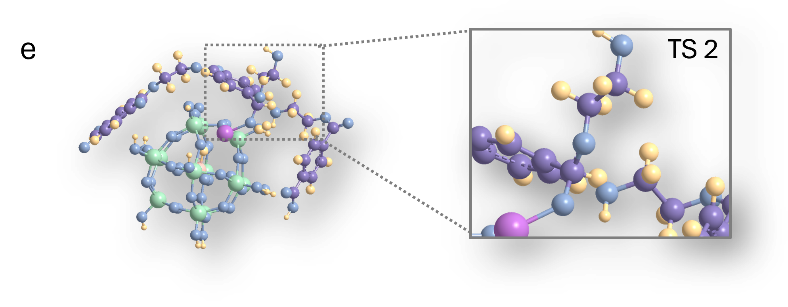


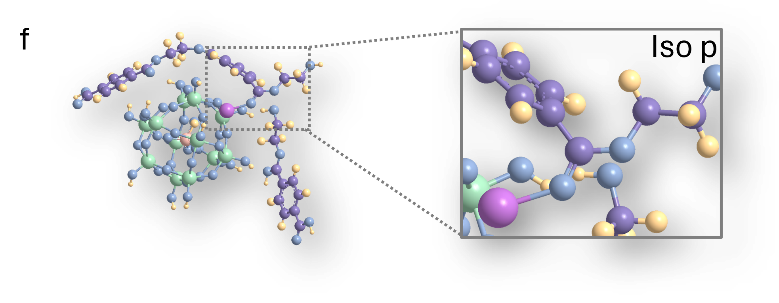


**Figure S26**. Schematic diagrams of the key reaction steps in catalytic depolymerization of PET by Pd-CsPTA.


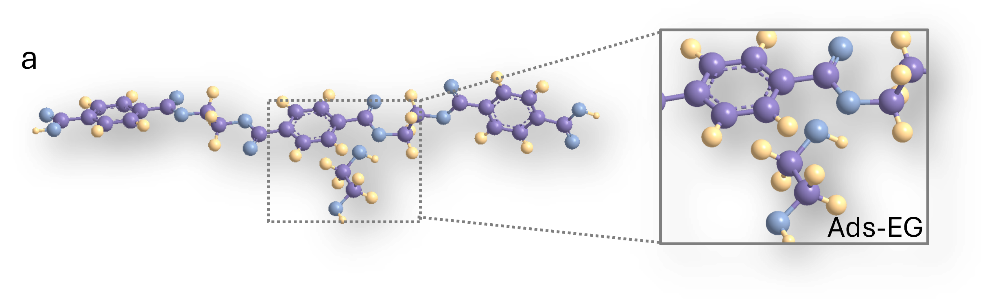


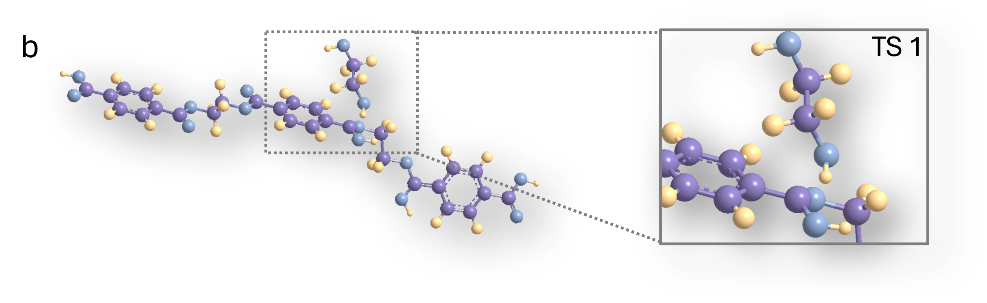


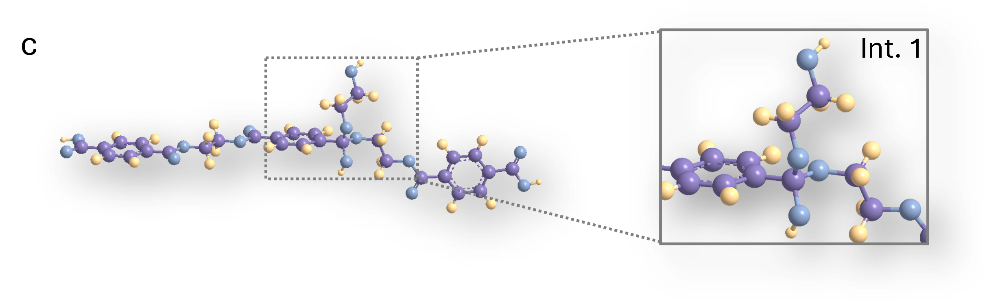


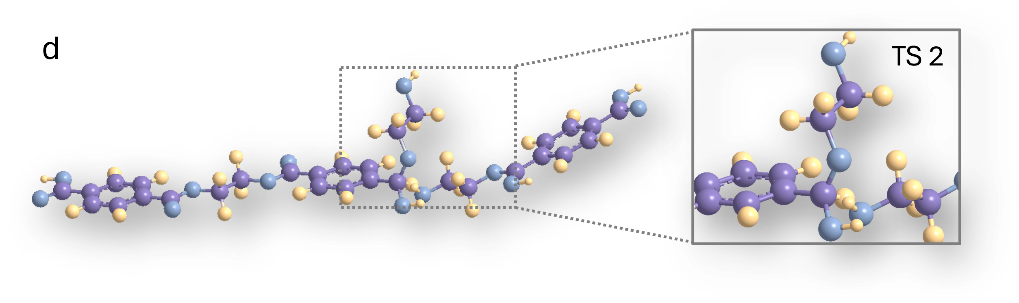


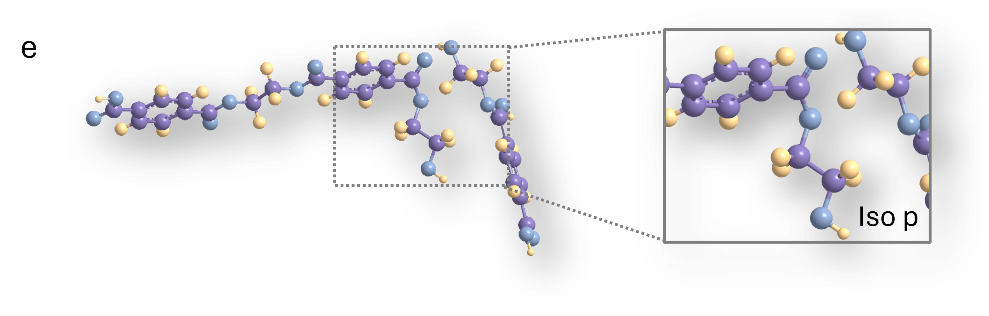


**Figure S27**. Schematic diagrams of the key reaction steps in depolymerization of PET without Pd-CsPTA.

In the absence of Pd-CsPTA, the hydroxyl oxygen of EG interacted with the carbonyl carbon of PET at high temperatures, forming TS 1 (Figure S22b). Subsequently, the oxygen atom of EG underwent nucleophilic attack by the carbonyl carbon, generating Int.1 (Figure S22c). Unlike catalytic reactions, this process did not involve the interaction between the Pd-CsPTA and PET, resulting in lower reactivity of the carbonyl carbon, thus requiring elevated temperatures for activation.

**Table S1**. EXAFS curve-fitting parameters at the Pd K-edge for Pd-foil, PdO and Pd-CsPTA.

|  | **Path** | **S_0_^2^** | **σ^2^ (Å^2^)** | **R (Å)** | **CN** |
| --- | --- | --- | --- | --- | --- |
| Pd-foil | Pd-Pd | 0.78 ± 0.05 | 0.004 ± 0.000 | 2.738 ± 0.020 | 12 |
| PdO | Pd-O | 0.78 ± 0.06 | 0.002± 0.001 | 2.030 ± 0.001 | 4 |
| Pd-CsPTA | Pd-O | 0.78 ± 0.03 | 0.005 ± 0.001 | 2.004 ± 0.060 | 3.7 ± 0.2 |

S_0_^2^ is amplitude reduction factor; σ^2^ is Debye-Waller factor; R is interatomic distance between central atoms and surround coordination atoms; CN is the coordination number.

**Table S2**. Structural optimization results of the Pd coordination site.

| **Name** | **Coordination mode** | **Coordination number** | **Binding energy (kcal/mol)** |
| --- | --- | --- | --- |
| Site-1 | four-fold oxygen hollow site | 4 | -64.22 |
| Site-2 | three-fold oxygen hollow site | 3 | -43.09 |

**Table S3**. Specific surface area, pore volume and pore size of AC and Pd-CsPTA/AC.

|  | **Specific surface area (m²/g)** | **Pore Volume (cc/g)** | **Average pore size (nm)** |
| --- | --- | --- | --- |
| AC | 49.2 | 0.191 | 3.73 |
| Pd-CsPTA/AC | 32.4 | 0.102 | 3.54 |

**Table S4**. A comparison of catalysts for PET depolymerization.

| Catalysts | Temperature | m(EG)/g | m(PET)/g | m(Catalyst)/g | time/h | C_PET_ (%) | Refs. |
| --- | --- | --- | --- | --- | --- | --- | --- |
| MeONa&EtONa | 160 | 10 | 1 | 0.04 | 2 | 5 | ^[7]^ |
|  | 170 |  |  |  |  | 10 |  |
| [C_4_mim]_2_[OAc] | 160 | 10 | 1 | 0.1 | 1 | 30 | ^[8]^ |
| [Bmim]_2_[CoCl]_4_ | 160 | 20 | 5 | 1 | 3 | 11 | ^[9]^ |
| Zn (OAc)_2_ | 180 | 38.75 | 10 | 22.5 | 6 | 44 | ^[10]^ |
| DMICA | 170 | 20 | 1 | 0.4 | 4 | 51 | ^[8]^ |
| ZnO-Fe_3_O_4_ | 170 | 60 | 10 | 0.1 | 1.5 | 75 | ^[11]^ |
| MAF-6 | 180 | 30 | 5 | 0.05 | 4 | 92 | ^[12]^ |
| KOAc | 150 | 38.75 | 10 | 22.5 | 6 | 15 | ^[8]^ |
| ZnCl_2_ | 180 | 25 | 1 | 0.04 | 24 | 75 | ^[13]^ |
| MnO_x_ | 180 | 20 | 1 | 0.02 | 3 | 43 | ^[14]^ |
| TiO_2_ | 180 |  |  |  |  | 56 |  |
| CeO_2_ | 180 |  |  |  |  | 45 |  |
| DES | 170 | 25 | 5 | 0.025 | 1 | 19 | ^[15]^ |
| SiO_2_-Fe_2_O_3_-NH_2_ SB | 180 | 5 | 1 | 0.025 | 3 | 41 | ^[16]^ |
| This work | 150 | 3 | 0.2 | 0.012 | 4 | 37 |  |
|  | 160 |  |  |  |  | 66 |  |
|  | 170 |  |  |  |  | 83 |  |
|  | 180 |  |  |  |  | 100 |  |

**Table S5**. Binding energy between Pd-CsPTA and PET through the different sites on PET.

|  | **Adsorption site** | **Binding Energy** |
| --- | --- | --- |
| Site-1 | Double bond oxygen in carbonyl group | -59.05 |
| Site-2 | Single bond oxygen in carbonyl group | -40.93 |
| Site-3 | Benzene ring | -47.57 |

Reference

[1] M. J. Frisch; G. W. Trucks; H. B. Schlegel; G. E. Scuseria; M. A. Robb; J. R. Cheeseman; G. Scalmani; V. Barone; G. A. Petersson; H. Nakatsuji; X. Li; M. Caricato; A. V. Marenich; J. Bloino; B. G. Janesko; R. Gomperts; B. Mennucci; H. P. Hratchian; J. V. Ortiz; A. F. Izmaylov; J. L. Sonnenberg; Williams; F. Ding; F. Lipparini; F. Egidi; J. Goings; B. Peng; A. Petrone; T. Henderson; D. Ranasinghe; V. G. Zakrzewski; J. Gao; N. Rega; G. Zheng; W. Liang; M. Hada; M. Ehara; K. Toyota; R. Fukuda; J. Hasegawa; M. Ishida; T. Nakajima; Y. Honda; O. Kitao; H. Nakai; T. Vreven; K. Throssell; J. A. Montgomery Jr.; J. E. Peralta; F. Ogliaro; M. J. Bearpark; J. J. Heyd; E. N. Brothers; K. N. Kudin; V. N. Staroverov; T. A. Keith; R. Kobayashi; J. Normand; K. Raghavachari; A. P. Rendell; J. C. Burant; S. S. Iyengar; J. Tomasi; M. Cossi; J. M. Millam; M. Klene; C. Adamo; R. Cammi; J. W. Ochterski; R. L. Martin; K. Morokuma; O. Farkas; J. B. Foresman; D. J. Fox Gaussian 16 Rev. C.01, Wallingford, CT, **2016**.

[2] C. Adamo; V. Barone, Toward reliable density functional methods without adjustable parameters: The PBE0 model. *J. Chem. Phys.* **1999,** *110* (13), 6158-6170.

[3] S. Grimme; S. Ehrlich; L. Goerigk, Effect of the Damping Function in Dispersion Corrected Density Functional Theory. *J. Comput. Chem.* **2011,** *32* (7), 1456-1465.

[4] F. Weigend; R. Ahlrichs, Balanced basis sets of split valence, triple zeta valence and quadruple zeta valence quality for H to Rn: Design and assessment of accuracy. *Phys. Chem. Chem. Phys.* **2005,** *7* (18), 3297-3305.

[5] F. Weigend, Accurate Coulomb-fitting basis sets for H to Rn. *Phys. Chem. Chem. Phys.* **2006,** *8* (9), 1057-1065.

[6] A. V. Marenich; C. J. Cramer; D. G. Truhlar, Universal Solvation Model Based on Solute Electron Density and on a Continuum Model of the Solvent Defined by the Bulk Dielectric Constant and Atomic Surface Tensions. *J. Phys. Chem. B* **2009,** *113* (18), 6378-6396.

[7] S. Javed; D. Vogt, Development of Eco-Friendly and Sustainable PET Glycolysis Using Sodium Alkoxides as Catalysts. *ACS Sustainable Chem. Eng.* **2023,** *11* (31), 11541-11547.

[8] Z. Wang; Y. Jin; Y. Wang; Z. Tang; S. Wang; G. Xiao; H. Su, Cyanamide as a Highly Efficient Organocatalyst for the Glycolysis Recycling of PET. *ACS Sustainable Chem. Eng.* **2022,** *10* (24), 7965-7973.

[9] Q. Wang; Y. Geng; X. Lu; S. Zhang, First-Row Transition Metal-Containing Ionic Liquids as Highly Active Catalysts for the Glycolysis of Poly(ethylene terephthalate) (PET). *ACS Sustainable Chem. Eng.* **2015,** *3* (2), 340-348.

[10] N. H. Le; T. T. Ngoc Van; B. Shong; J. Cho, Low-Temperature Glycolysis of Polyethylene Terephthalate. *ACS Sustainable Chem. Eng.* **2022,** *10* (51), 17261-17273.

[11] L.-X. Yun; Y. Wei; Q. Sun; Y.-T. Li; B. Zhang; H.-T. Zhang; Z.-G. Shen; J.-X. Wang, Magnetic hollow micro-sized nanoaggregates for synergistically accelerating PET glycolysis. *Green Chem.* **2023,** *25* (17), 6901-6913.

[12] R.-X. Yang; Y.-T. Bieh; C. H. Chen; C.-Y. Hsu; Y. Kato; H. Yamamoto; C.-K. Tsung; K. C. W. Wu, Heterogeneous Metal Azolate Framework-6 (MAF-6) Catalysts with High Zinc Density for Enhanced Polyethylene Terephthalate (PET) Conversion. *ACS Sustainable Chem. Eng.* **2021,** *9* (19), 6541-6550.

[13] Y. Yang; S. Sharma; C. Di Bernardo; E. Rossi; R. Lima; F. S. Kamounah; M. Poderyte; K. Enemark-Rasmussen; G. Ciancaleoni; J.-W. Lee, Catalytic Fabric Recycling: Glycolysis of Blended PET with Carbon Dioxide and Ammonia. *ACS Sustainable Chem. Eng.* **2023,** *11* (30), 11294-11304.

[14] B. Swapna; N. Singh; S. Patowary; P. Bharali; G. Madras; P. Sudarsanam, Efficient glycolysis of used PET bottles into a high-quality valuable monomer using a shape-engineered MnOx nanocatalyst. *Catal. Sci. Technol.* **2024,** *14* (19), 5574-5587.

[15] F. Li; X. Yao; R. Ding; Y. Bao; Q. Zhou; D. Yan; Y. Li; J. Xu; J. Xin; X. Lu, Directional glycolysis of waste PET using deep eutectic solvents for preparation of aromatic-based polyurethane elastomers. *Green Chem.* **2024,** *26* (18), 9802-9813.

[16] E. Casey; R. Breen; J. S. Gomez; A. P. M. Kentgens; G. Pareras; A. Rimola; J. D. Holmes; G. Collins, Ligand-Aided Glycolysis of PET Using Functionalized Silica-Supported Fe(2)O(3) Nanoparticles. *ACS Sustain. Chem. Eng.* **2023,** *11* (43), 15544-15555.
